# Synthesis of Tronocarpine Framework via a Michael/Lactamization/Michael Cascade Process: Synthesis of Dihydrotronocarpine

**DOI:** 10.1021/acs.joc.5c03062

**Published:** 2026-04-06

**Authors:** Mario Castañón-Garcíaa, Carlos H. Escalantea, Luis D. Mirandaa

**Affiliations:** Instituto de Química, Circuito Exterior S.N., Ciudad Universitaria, Universidad Nacional Autónoma de México, Coyoacán, Ciudad de México 04510, México

## Abstract

This paper describes an efficient strategy that uses a Michael/lactamization/Michael one-pot cascade process to construct the pentacyclic core of tronocarpine. This key transformation used a seven-membered lactam derived from tryptamine and a Michael double acceptor to produce an advanced intermediate with all of the necessary functional groups to synthesize the natural product in 53% yield. Dihydrotronocarpine was then synthesized from this intermediate in two additional steps.

## Graphical abstract

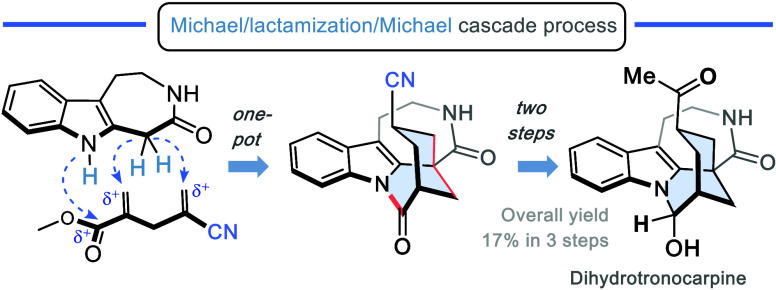

## Introduction

The genus *Tabernaemontana* encompasses over 100 plant species and is notable for producing a wide variety of structurally diverse indole alkaloids,^1,2^ which include the structurally novel subgroup chippiine–dippinine alkaloids.^3^ In this regard, in 2000, Kam et al.^4^ isolated tronocarpine from *Tabernaemontana corymbosa*, a Malayan plant. This alkaloid has a distinctive molecular structure consisting of five fused rings, a seven-membered lactam, and an all-carbon quaternary center. It also contains an azabicyclic system with a hemiaminal system, a conjugated enone system, and three chiral centers. From a pharmacological perspective, tronocarpine has demonstrated significant cytotoxic activity against cells resistant to NF-κB and vincristine. Its potential to reverse multidrug resistance also makes it a promising candidate for therapeutic development.^5^

Since its isolation, several methodologies for synthesizing indole-fused derivatives, which form the core of tronocarpine, have been reported. Among these, our group reported a three-step strategy in 2009 to construct the tetracyclic system **5** based on a Michael/lactamization reaction cascade (Scheme 1a).^6c^ Then, in 2014, Martínez and coworkers^7^ reported a synthetic approach to the pentacyclic core in seven steps via a Michael/aldol cascade reaction between **2** and an α,β-unsaturated aldehyde (derived from δ-valerolactone). Five years later, in 2019, the group of Fu-She Han^8^ reported the first asymmetric total synthesis and absolute configuration determination of (+)-tronocarpine. The synthetic route consisted of 20 steps with an overall yield of 2.2%. The key transformation involved a Michael/aldol cascade reaction between nitrile **6**, acrolein, and a chiral cinchona-derived phase-transfer catalyst, affording a tetracyclic system, which was advanced to the natural product (Scheme 1b). One year later, the same group published an updated version of their approach, reducing the number of steps from 20 to 11 and increasing the overall yield to 10.8%.^9^ In the same year, Namba and coworkers reported a racemic total synthesis via a one-pot construction of the pentacyclic skeleton from indole-substituted **8**.^10^ This tandem cyclization involved the formation of an α,β-unsaturated aldehyde, an intramolecular aldol reaction, six- and seven-membered lactamizations, and azide reduction. The complete synthetic route comprised seven reaction steps with an overall yield of 1.87% (Scheme 1d).

**Scheme 1:**
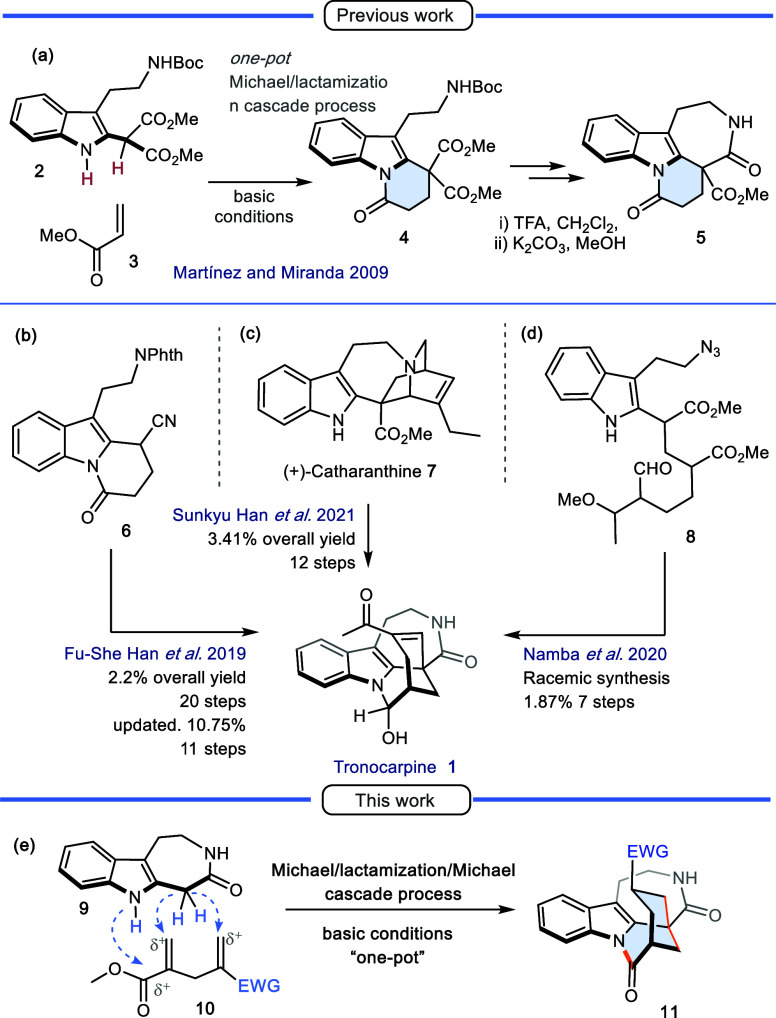
Synthesis of Tronocarpine: Previous and Present Work

The most recent synthesis, published in 2021 by Han^11^ et al., involved the ring-opening functionalization of a tertiary amine to synthesize various alkaloids, starting with (+)-catharanthine (**7**). A ring-opening reaction was the key feature in a 12-step synthesis of (−)-tronocarpine, with an overall yield of 3.41% (Scheme 1c).

In the present study, we envisaged the retrosynthetic plan illustrated in Scheme 2. First, the double bond of the enone system would be introduced in the final stage through a dehydrogenation process.^12^ Thus, the ketone in the intermediate could be obtained by conversion of a nitrile into the methylketone,^13^ and the hemiaminal group could be formed by chemoselective reduction of the corresponding lactam. The strategy for synthesizing the pentacyclic intermediate **11** relied on a one-pot Michael/lactamization/Michael cascade process involving the known compounds tryptamine-derived lactam^6c^ **9** and Michael double acceptor^14^ **10** (Scheme 1e).

**Scheme 2:**
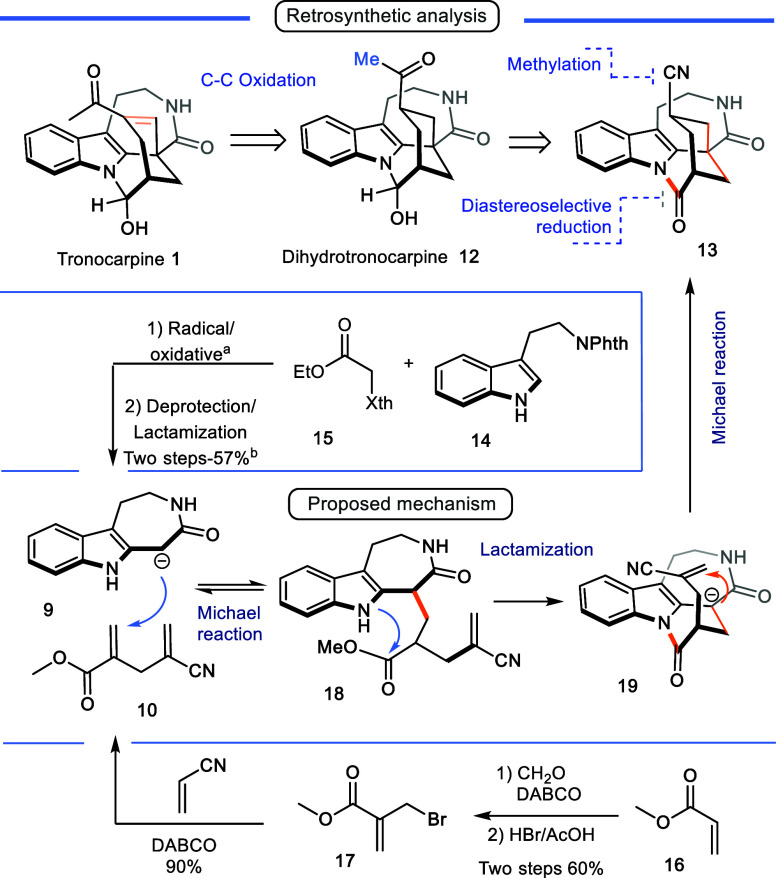
Retrosynthetic Analysis, Proposed Mechanism, and Starting Materials Synthesis^a^

a DLP, 1,2-DCE, 84 °C, 12 h, 71%, ^b^MeOH, aq. methylamine (2 M), 90 °C, 24 h, 80%.

## Results and Discussion

Thus, the synthesis commenced with the synthesis of the tryptamine-derived lactam **9** according to the reported methodology with a slight modification.^5c^ Phthalimide-protected tryptamine **14** (instead of the reported N-Boc protected)^5,6^ was C-2 alkylated with the xanthate **15** via an oxidative radical addition. Removing the phthalimide group with concomitant lactamization generated lactam **9** in 40% overall yield in two steps (Scheme 2). On the other hand, synthesizing the double Michael acceptor **10** involved obtaining methyl 2-(bromomethyl)­acrylate **17** through a Baylis–Hillman reaction using methyl acrylate and formaldehyde, followed by bromination. A second Baylis–Hillman reaction with acrylonitrile then generated the desired product **10** with an overall yield of 54% in the three steps (Scheme 2).^14^

Thus, with the starting materials in hand, we began the synthesis by optimizing the Michael-lactamization-Michael cascade process. In principle, the proposed mechanism starts with the deprotonation of lactam **9** under basic conditions to generate the corresponding enolate. This enolate then undergoes the first 1,4-Michael addition with acceptor **10** to form ester **18**, which collapses to tetracyclic **19** via lactamization. A second carbanion is then formed, followed by an intramolecular 1,4-Michael addition, which finally assembles the complete pentacyclic system in a single reaction step.

Thus, in the first experiment, lactam **9** and the double Michael acceptor **10** were combined in acetonitrile using benzyltriethylammonium chloride (TEBA) as a phase-transfer catalyst (PTC) and potassium carbonate as the base. However, after 17 h under these conditions, compound **13** was obtained in trace amounts (Table 1, entry 1). The low solubility of lactam **9**, which was observed, might likely contribute to the low yield. We evaluated various solvents, temperatures, phase-transfer agents, and bases to improve the outcome. Thus, when the reaction was carried out using a solvent with a high dielectric constant, such as dimethylformamide (DMF), at 82 °C, the yield increased to 18% (entry 2). However, using dimethyl sulfoxide (DMSO) did not improve the outcome (entry 3). Reactions conducted at room temperature showed no conversion. On the other hand, replacing the phase-transfer agent with 1.5 equiv of benzyltrimethylammonium hydroxide (Triton B) and 9 equiv of potassium carbonate resulted in a yield of 13% (entry 4). Incidentally, Triton B is known to function as both a phase-transfer agent and a base (i.e., a source of hydroxyl groups). To investigate whether the acid–base reaction had been facilitated by the Triton B or the K_2_CO_3_, a test was conducted using only 2.2 equiv of Triton B for 17 h. Under these conditions, the yield was increased to 26% (entry 5) despite the absence of an inorganic base. Further attempts to improve the yield by testing bases such as *t*-BuOK and sodium hydroxide (entries 6 and 7) were not successful. Based on these results, Triton B was selected as the base for subsequent optimization.

**Table 1:**
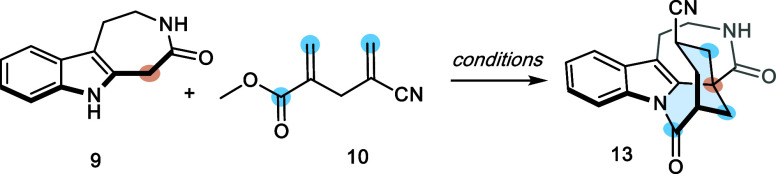
Optimization of Reaction Conditions

Entry	10 (equiv)	Solvent (0.05 M)	PTC (equiv)	Base	t (h)	Yield (%)
1^a^	1.5	MeCN	TEBA	K_2_CO_3_	17	Traces
2^a^	1.5	DMF	TEBA	K_2_CO_3_	17	18
3^a^	1.5	DMSO	TEBA	K_2_CO_3_	17	Traces
4^a^	1.5	DMF	Triton B	K_2_CO_3_	17	13
5^b^	3	DMF	Triton B	—	17	26
6	3	DMF	—	NaOH (2.2)	17	0
7	1.5	DMSO	—	*t*-BuOK (2.2)	3	Traces
8^b^	3	DMF	Triton B	—	3.5	39
9^b^	4	DMF	Triton B	—	3.5	53
10^b^,^c^	10	DMF	Triton B	—	17	37

a 1.5 equiv of PTC and 9 equiv of base.

b 2.2 equiv of PTC.

c Heating MW, all the experiments were conducted at 82 °C.

Next, the reaction time and the equivalents of Michael double acceptor **10** were optimized. In Experiment 8, we added 2.2 equiv of Triton B in five portions at 30 min intervals, which resulted in an increased yield of 39%. The same approach was used to add four equivalents, one per hour, of the double acceptor. Satisfactorily, the pentacyclic product was isolated in 53% yield under these conditions (entry 9). However, increasing the equivalents of the double acceptor to ten resulted in a yield of 37%.

We concluded that the best results for the Michael/lactamization/Michael cascade were achieved by initially using two equivalents of compound **10** (with an additional equivalent added every hour for a total of 4 equiv added during a total reaction time of 3.5 h), one equivalent of lactam **9** in DMF at 82 °C, and 2.2 equiv of Triton B added in five portions at 30 min intervals. Notably, this cascade reaction begins with relatively simple starting materials and forms three new bonds (two C–C and one C–N) and three chiral centers in a diastereoselective manner, assembling directly the complete pentacyclic backbone. It is worth noting that the intermediate can be further transformed into the natural product by adjusting the present functional groups, i.e., diastereoselective reduction of the carbonyl group and transformation of the cyano group into the necessary enone.

Thus, with pentacyclic **13** in hand, the next step in synthesizing tronocarpine involved the chemo- and diastereoselective reduction of the more electrophilic amide in the *N*-acylindol fragment to obtain hemiaminal **20a** (α–OH). Interestingly, using lithium triethylborohydride (Super-Hydride) at −41 °C resulted in the formation of the β-hydroxyl isomer **20b** (β–OH), yielding 76%. In contrast, switching to a bulkier reducing agent, potassium tri-*sec*-butylborohydride (K-Selectride), favored the formation of the α-hydroxyl isomer **20a** (α–OH, 97:3 dr) in 60% yield. However, since the natural product contains the α-epimer, synthesis continued using this isomer (Scheme 3).

**Scheme 3:**
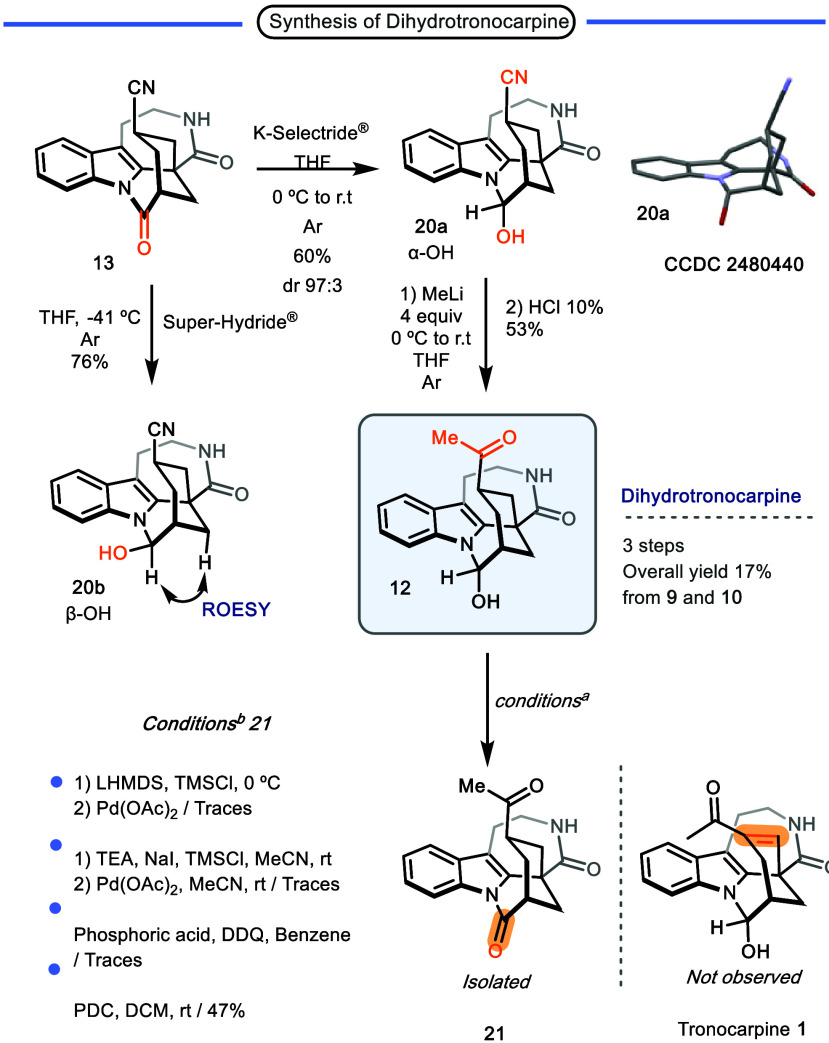
Synthesis of Dihydrotronocarpine^a^ ^b^

a Complete conditions are provided in the SI.
b These conditions afforded compound 21.

Subsequently, we converted the nitrile group into the corresponding methylketone. In the first attempt, no reaction was observed using methylmagnesium bromide. Satisfactorily, when it was replaced with methyllithium, the desired ketone **12** was successfully obtained, with a yield of 53% (Scheme 3).

As proposed originally, the final reaction step involved C–C dehydrogenation to form the enone present in the natural product. Traditional Saegusa–Ito conditions were initially proposed to perform this transformation, but unfortunately, the expected enone was not obtained under these conditions. Increasing the temperature to favor the thermodynamic enolate also failed to achieve its formation. Conversely, we observed the oxidation of the hemiaminal group to form the corresponding amide **21**. No product formation was observed using the oxidation conditions reported by the group of Nicolaou^15^ to obtain α,β-unsaturated carbonyl compounds using IBX. Also, when a primary amine such as benzylamine was combined with palladium acetate in DMSO at 75 °C under an oxygen atmosphere, oxidation of the hemiaminal to the corresponding amide **21** was only observed.^16^ Finally, a report by List et al.^17^ described the formation of α,β-unsaturated compounds using phosphoric acid and DDQ. Unfortunately, when compound **12** was subjected to these conditions, unwanted compound **21** was again the only product observed. Additionally, the selective oxidation of α-hydroxyl isomer **12** was achieved with PDC to afford the amide **21** in 47% yield.^18^ At this stage, we decided not to pursue the synthesis of tronocarpine further. However, although total synthesis could not be achieved, the method reported herein for the rapid assembly of the complete pentacyclic system paves the way for synthesizing alkaloid analogues in medicinal chemistry programs.

## Conclusions

In conclusion, an efficient methodology for constructing the pentacyclic framework of the tronocarpine natural alkaloid, from lactam **9** and Michael double acceptor **10**, was developed. This Michael/lactamization/Michael cascade reaction has several noticeable features: a) it starts with relatively simple starting materials; b) it forms three new bonds (two C–C and one C–N) and three chiral centers (one of them an all-carbon quaternary center) in a diastereoselective manner; c) it directly assembles the complete pentacyclic skeleton **13**; and d) the product contains functional groups suitable for its transformation into the natural product. Diastereoselective reduction of intermediate lactam **13** using K-Selectride subsequently afforded the hemiaminal **20a** in 60% yield, and finally, methylation of this intermediate with methyllithium then furnished the methylketone **21** in 53% yield. Thus, the diastereoselective synthesis of dihydrotronocarpine was accomplished in three steps with an overall yield of 17% from **9** and **10**.

## Safety Issues

### Caution

Solution of lithium triethylborohydride (Super-Hydride) reacts violently with water and may ignite in moist air. Commercial solutions (1.0 M in THF) are flammable. Exposure to moisture or air must be strictly avoided. Superhydride should be handled only under an inert atmosphere by individuals trained in its safe and proper use, using appropriate protective equipment.

### Caution

Potassium tri-*sec*-butylborohydride (K-Selectride) reacts violently with water and may ignite in moist air. Commercial solutions (1.0 M in THF) are flammable. Exposure to moisture or air must be strictly avoided. Superhydride should be handled only under an inert atmosphere by individuals trained in its safe and proper use, using appropriate protective equipment.

### Caution

Methyllithium reacts violently with water and may ignite in moist air. Commercial solutions (1.6 M in diethyl ether) are flammable. In contact with water, it releases methane , which can ignite spontaneously. Contact with air or moisture must be strictly avoided. MeLi should be handled under an inert atmosphere by properly trained personnel wearing suitable personal protective equipment.

## Supplementary Material



## Data Availability

The data underlying this study are available in the published article and its Supporting Information

## Notes

Experimental procedures, ^1^H and ^13^C NMR spectra of products, and X-ray crystallographic information (PDF)
