# Supplementary material for: Synthesis of Tronocarpine Framework via a Michael/Lactamization/Michael Cascade Process: Synthesis of Dihydrotronocarpine
Source: J Org Chem. 2026 Apr 6;91(15):5293–7. doi: 10.1021/acs.joc.5c03062 (PMC13097250; doi:10.1021/acs.joc.5c03062)
Supplement: Supplementary file 1 [file jo5c03062_si_001.pdf]

Supporting Information

Synthesis of Tronocarpine Framework via a Michael/Lactamization/Michael Cascade Process: Synthesis of Dihydrotronocarpine

Mario Castañón-García[a], Carlos H. Escalante[a], and Luis D. Miranda[a*].

[a]*Instituto de Química, Universidad Nacional Autónoma de México, Circuito Exterior S.N., Ciudad Universitaria, Coyoacán, Ciudad de México, 04510, Mexico.*

*Email: lmiranda@unam.mx

Table of Contents

1. ***General information*** **S2**

2. ***Experimental procedures*** **S3**

2.1 Procedures for the synthesis of substrate S6 .... **S3**

2.2 Procedures for the synthesis of pentacyclic compound 13. .... **S4**

2.3 Procedures for the synthesis of hemiaminal. **S5**

2.3.1 Procedures for the synthesis of β-hydroxy-hemiaminal 20b. **S6**

2.3.2 Procedures for the synthesis of α-hydroxy-hemiaminal 20a. **S6**

2.4 Procedures for the synthesis of ketone 12. .... **S7**

2.5 Procedures for the synthesis of 21. .... **S7**

3. ***Experimental procedures*** **S9**

4. ***X-Ray Crystallographic Data*** **S19**

5. ***References*** **S21**

1. General information

All chemicals and solvents were purchased from Sigma-Aldrich or Tecsiquim. Reactions progress was monitored by analytical thin layer chromatography (TLC) using silica gel 60 F254 plates purchased from Merck. Monitoring was achieved by shortwave UV light (254 nm). Chromatographic purification of products was accomplished by flash chromatography on silica gel technical grade (Merck, 230-400 mesh). Melting points were determined on a Fisher apparatus and are uncorrected. ^{1}H and $^{13}C\{^{1}H\}$ NMR spectra were recorded on a Jeol Eclipse-300 MHz, Bruker Avance III 400 MHz, 500 MHz and 700 MHz model spectrometers using DMSO-d_6, $CDCl_3$, MeOD and Acetone- d_6 as solvent. NMR coupling constants are reported in Hertz (Hz). Chemical shifts (δ) are reported in parts per million (ppm)and the residual solvents peak was used as an internal reference. Multiplicity was indicated as follows: s (singlet), d (doublet), t (triplet), q (quartet), p (quintet), m (multiplet), dd (doublet of doublet), bs (broad singlet). Coupling constants were reported in Hertz (Hz). The MS-DART spectra were obtained on a JEOL DART AccuTOF JMS-T100CC. X-ray diffraction studies were performed on a Bruker Smart APEX II CCD diffractometer with graphite-monochromatic Mo Kα irradiation. IR spectra were recorded on a Perkin Elmer 343 Frontier FT-IR spectrophotometer by attenuated total reflection (ATR-FTIR).

2. Experimental procedures

2.1 Procedures for the synthesis of substrate 9.

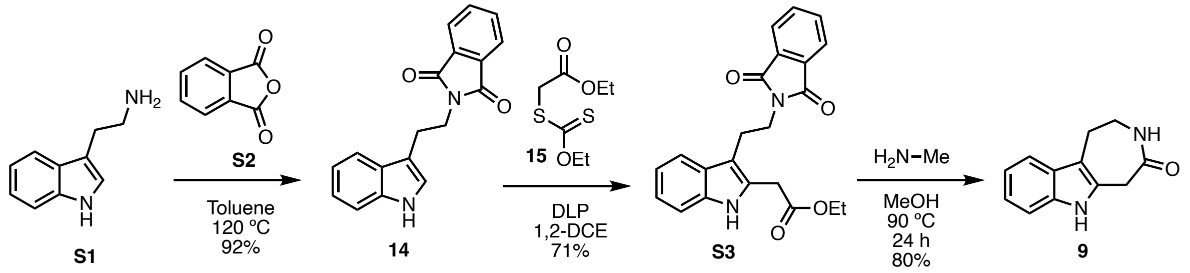

Scheme S1. Preparation of Substrate **9**.

In a round-bottom flask equipped with a magnetic stir bar, tryptamine (**S1**, 2.00 g, 12.23 mmol) and phthalic anhydride (**S2**, 2.02 g, 13.70 mmol) were dissolved in toluene (24.5 mL). The reaction mixture was stirred at 120 °C in a metal heating mantle for 24 h and then cooled to room temperature. The solvent was removed under reduced pressure, and the precipitate was filtered under vacuum. The solid was washed sequentially with ethyl ether to give **14**[1] (3.26 g, 11.22 mmol) as a brown solid. Yield: 92 %. **^{1}H NMR** (500 MHz, $CDCl_3$) δ 8.03 (s, 1H), 7.83 (dd, *J* = 5.5, 3.1 Hz, 2H), 7.74 (d, *J* = 7.9 Hz, 1H), 7.70 (dd, *J* = 5.5, 3.0 Hz, 2H), 7.35 (d, *J* = 8.1 Hz, 1H), 7.19 (ddd, *J* = 8.1, 7.0, 1.2 Hz, 1H), 7.13 (ddd, *J* = 8.0, 7.0, 1.1 Hz, 1H), 7.09 (d, *J* = 2.2 Hz, 1H), 4.05 – 3.98 (m, 2H), 3.17 (ddd, *J* = 8.6, 6.3, 0.9 Hz, 2H). **^{13}C{^{1}H} NMR** (125 MHz, $CDCl_3$) δ 168.5, 136.4, 134.0, 132.3, 127.5, 123.3, 122.3, 122.1, 119.6, 119.0, 112.6, 111.2, 38.7, 24.6.

To a solution of **14** (2.00 g, 6.88 mmol) in 1,2-dichloroethane (30 mL) was added xanthate **15** (2.58 g, 12.4 mmol). In a separate flask was added dilauroyl peroxide (DLP, 4.94 g, 18.4 mmol) in 1,2-dichloroethane (15 mL). Both solutions were deoxygenated, and the first flask was heated to 84 °C in a metal heating mantle. Subsequently, the DLP solution was added in five portions at 30 min intervals, and the reaction mixture was allowed to reflux for 12 h. The solvent was evaporated under reduced pressure, and the reaction crude was purified by column chromatography on silica gel (eluent: Hex:EtOAc, 7:3) to give **S3** (1.84 g, 4.88 mmol) as a yellow oil. Yield: 71%. **^{1}H NMR** (500 MHz, $CDCl_3$) δ 8.73 (s, 1H), 7.82 (dd, *J* = 5.5, 3.0 Hz, 2H), 7.73 – 7.65 (m, 3H), 7.30 (d, *J* = 7.9 Hz, 1H), 7.14 (ddd, *J* = 8.1, 7.0, 1.3 Hz, 1H), 7.09 (ddd, *J* = 8.1, 7.0, 1.1 Hz, 1H), 4.19 (q, *J* = 7.2 Hz, 2H), 3.95 – 3.89 (m, 2H), 3.85 (s, 2H), 3.14 – 3.07 (m, 2H), 1.29 (t, *J* = 7.1 Hz, 3H). **^{13}C{^{1}H} NMR** (125 MHz, $CDCl_3$) δ 170.7, 168.4, 135.7, 133.9, 132.3, 128.0, 127.7, 123.2, 122.0, 119.6, 118.5, 110.9, 109.6, 61.5, 38.4, 31.8, 23.4, 14.2. **HRMS** (DART) m/z calcd for $C_{22}H_{21}N_2O_4$ $[M + H]^+$: 377.1501, found 377.1488.

Finally, compound **S3** was dissolved in MeOH (50 mL), and aqueous methylamine (2.0 M, 1.9 mL) was added. The reaction mixture was heated at 90 °C in a metal heating mantle for 24 h. The mixture was cooled to room temperature, and the solvent was removed under reduced pressure. The residue was diluted with water and extracted with EtOAc (3 × 20 mL). The combined organic layers were dried over anhydrous Na_2SO_4, filtered, and the solvent was removed under reduced pressure. The reaction crude was purified by column chromatography on silica gel (eluent: DCM:MeOH, 9:1)

to give **9**[2] (0.78 g, 3.92 mmol) as a white solid. Yield: 80%.^{1}**H NMR** (500 MHz, DMSO-*d6*) (s, 1H), 7.77 (t, *J* = 6.5 Hz, 1H), 7.35 (d, *J* = 7.7 Hz, 1H), 7.27 (d, *J* = 8.0 Hz, 1H), 7.03 (ddd, *J* = 8.1, 6.9, 1.2 Hz, 1H), 6.98 – 6.94 (m, 1H), 3.78 (s, 2H), 3.54 (q, *J* = 6.2 Hz, 2H), 2.81 – 2.74 (m, 2H). ^{13}**C{^{1}H} NMR** (125 MHz, DMSO-*d6*) δ 172.4, 134.8, 128.4, 127.0, 120.7, 118.4, 117.3, 110.6, 108.1, 35.0, 25.6.

2.2 Procedures for the synthesis of pentacyclic compound 13.

Table S1. Optimization of Reaction Conditions for the Preparation of Pentacycle 13.

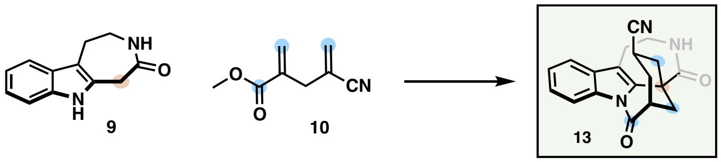

Entry	10 (equiv)	Solvent (0.05 M)	PTC	Base	Temperature	Time (hours)	Yield (%)
1	1.5	MeCN	TEBA (1.5 equiv)	K_2CO_3 (9 equiv)	82 °C	17	TRACES
2	1.5	DMF	TEBA (1.5 equiv)	K_2CO_3 (9 equiv)	82 °C	17	18
3	1.5	DMSO	TEBA (1.5 equiv)	K_2CO_3 (9 equiv)	82 °C	17	TRACES
4	1.5	DMF	Triton B (1.5 equiv)	K_2CO_3 (9 equiv)	82 °C	17	13
5	3	MeCN	Triton B (1.5 equiv)	K_2CO_3 (9 equiv)	82 °C	17	10
6	3	DMF	Triton B (2.2 equiv)	---	82 °C	17	26
7	3	DMF	---	NaOH (2.2 equiv)	82 °C	17	---
8	1.5	DMSO	---	t-BuOK (2.2 equiv)	82 °C	3	TRACES
9	1.5	DMSO	---	t-BuOK (2.2 equiv)	t.a	3	TRACES
10	1.5	THF	---	LiHMDS (2.2 equiv)	-78 °C	2	TRACES
11	3	Dry DMF	Triton B (2.2 equiv)	---	82 °C	17	21
12	3	DMF	Triton B (2.2 equiv)	---	82 °C MW	0.33	23
13	3	DMF	Triton B (2.2 equiv)	---	82 °C	3.5	39
14	2 + (1 equiv /h)	DMF	Triton B (2.2 equiv) 5 parts	---	82 °C	4.5	53

15	10	DMF	Triton B (2.2 equiv) 5 parts	---	100 °C MW	17	37
16	6	DMF	Triton B (2.2 equiv) 10 parts	---	82 °C	7	TRACES
17	6	Toluene	Triton B (2.2 equiv) 10 parts	---	110 °C	7	12

In a round-bottom flask equipped with a magnetic stir bar, lactam **9** (0.10 g, 0.50 mmol, 1.0 equiv) and Michael's double acceptor **10** (0.151 g, 1.00 mmol, 2.0 equiv; plus 1.0 equiv added per hour) were dissolved in DMF (10 mL, 0.05 M). The reaction mixture was heated to 82 °C using a metal heating mantle, and benzyltrimethylammonium hydroxide (Triton B, 0.52 mL, 40% in MeOH, 1.15 mmol, 2.3 equiv) was added in five portions at 30 min intervals. After the final addition, the reaction mixture was stirred at 82 °C for an additional 1 h. Finally, brine was added and extracted with EtOAc (3 × 30 mL). The combined organic phase was dried over Na_2SO_4, filtered and the solvent was evaporated under reduced pressure. The residue was purified by column chromatography 99:1 DCM:MeOH to give **13** (0.085g, 0.266 mmol) as a white solid, m.p 280 °C (dec). Yield: 53 %. **^{1}H NMR** (700 MHz, $CDCl_3$) δ 8.51 (d, *J* = 8.2 Hz, 1H), 7.45 (d, *J* = 7.6 Hz, 1H), 7.41 (ddd, *J* = 8.3, 7.2, 1.3 Hz, 1H), 7.34 (td, *J* = 7.5, 1.0 Hz, 1H), 6.30 (t, *J* = 7.0 Hz, 1H), 3.88 (dddd, *J* = 15.1, 12.6, 5.8, 2.6 Hz, 1H), 3.57 (ddt, *J* = 15.2, 7.2, 3.6 Hz, 1H), 3.22 (p, *J* = 3.3 Hz, 1H), 3.05 (dt, *J* = 17.1, 2.8 Hz, 1H), 3.00 (dd, *J* = 12.7, 4.0 Hz, 1H), 2.69 (ddt, *J* = 12.9, 4.1, 2.0 Hz, 1H), 2.63 (tt, *J* = 13.0, 4.1 Hz, 1H), 2.53 (dddt, *J* = 13.7, 4.7, 3.3, 1.6 Hz, 1H), 2.43 (ddt, *J* = 14.0, 3.8, 1.8 Hz, 1H), 2.39 (dd, *J* = 14.0, 2.8 Hz, 1H), 2.18 (t, *J* = 12.9 Hz, 1H), 2.07 (td, *J* = 13.4, 4.6 Hz, 1H). **^{13}C{^{1}H} NMR** (175 MHz, $CDCl_3$) δ 173.5, 170.2, 134.5, 129.8, 129.3, 126.4, 124.8, 120.3, 119.0, 118.4, 116.8, 43.4, 40.2, 39.0, 38.9, 33.7, 31.2, 26.1, 23.7. **HRMS** (DART) m/z calcd for $C_{19}H_{18}N_3O_2$ $[M + H]^+$: 320.1399, found 320.1399. **FT-IR** (ATR): ν (cm^{-1}) 3214, 2240, 2221, 1690, 1611, 1457, 1375, 1327, 1193, 1018, 964, 747, 697.

2.3 Procedures for the synthesis of hemiaminal.

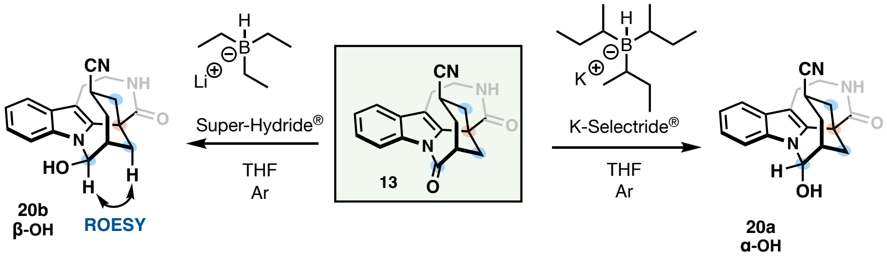

Scheme S2. Synthesis of Hemiaminals **20a** and **20b**.

2.3.1 Procedures for the synthesis of β-hydroxy-hemiaminal 20b.

To a solution of **13** (0.092 g, 0.028 mmol) in dry THF (5.8 mL) under an argon atmosphere was added dropwise, via syringe, lithium triethylborohydride (Super-Hydride®, 1.0 M in THF, 0.8 mL, 0.08 mmol) at –41 °C. Caution: this reagent is highly moisture- and air-sensitive and reacts violently with water; appropriate safety precautions should be taken. After 8 h, the reaction mixture was quenched with a saturated solution of ammonium chloride and extracted with DCM (3 × 30 mL). The combined organic phase was dried over Na_2SO_4, filtered and the solvent was removed under reduced pressure. The reaction crude was purified by column chromatography 97:3 DCM:MeOH to give **20b** (0.071 g, 0.22 mmol) as a white solid, m.p 135-138 °C. Yield: 76%. Diastereomeric ratio 97:3. **^{1}H NMR** (500 MHz, DMSO-d_6) δ 8.02 – 7.93 (m, 1H), 7.72 (d, *J* = 8.2 Hz, 1H), 7.43 (d, *J* = 7.8 Hz, 1H), 7.13 (t, *J* = 7.6 Hz, 1H), 7.05 (t, *J* = 7.4 Hz, 1H), 6.64 (d, *J* = 7.7 Hz, 1H), 5.78 (t, *J* = 7.0 Hz, 1H), 3.79 (td, *J* = 13.7, 12.9, 5.6 Hz, 1H), 3.30 (ddd, *J* = 11.2, 7.9, 3.9 Hz, 1H), 2.94 (d, *J* = 14.4 Hz, 1H), 2.77 – 2.65 (m, 3H), 2.54 (p, *J* = 3.3 Hz, 1H), 2.49 (s, 1H), 2.18 (dd, *J* = 13.9, 3.8 Hz, 1H), 1.91 (t, *J* = 12.8 Hz, 1H), 1.82 (d, *J* = 13.5 Hz, 1H), 1.72 (td, *J* = 13.1, 4.2 Hz, 1H). **^{13}C{^{1}H} NMR** (125 MHz, DMSO-d_6) δ 173.6, 135.9, 130.6, 127.8, 122.2, 121.6, 119.4, 117.9, 112.3, 110.3, 77.9, 42.8, 37.9, 32.8, 32.4, 27.3, 25.9, 23.1. **HRMS** (DART) m/z calcd for $C_{19}H_{20}N_3O_2$ $[M + H]^+$: 322.1555, found 322.1562. **FT-IR** (ATR): ν (cm^{-1}) 3360, 3056, 2241, 1643, 1462, 1357, 1293, 1235, 1151, 1086, 1022, 745, 684. Structural assignments were confirmed with additional 2D NMR data (gHSQC, and gROESY). Crystals of 20a were grown from a chloroform solution by slow evaporation at room temperature.

2.3.2 Procedures for the synthesis of α-hydroxy-hemiaminal 20a.

To a solution of **13** (0.096 g, 0.30 mmol) in dry THF (6 mL) under an argon atmosphere was added dropwise, via syringe, potassium tri-*sec*-butylborohydride (K-Selectride®, 1.0 M in THF, 2.40 mL, 2.40 mmol) at 0 °C. Caution: this reagent is highly moisture- and air-sensitive and reacts violently with water; appropriate safety precautions should be taken. After the addition, the reaction mixture was allowed to heat to room temperature and stirred for 7 h. The reaction mixture was quenched with saturated solution of ammonium chloride and extracted with DCM (3 × 30 mL). The combined organic phase was dried over Na_2SO_4, filtered and the solvent was removed under reduced pressure. The reaction crude was purified by column chromatography 97:3 DCM:MeOH to give **20a** (0.059 g, 0.28 mmol) as a white solid, m.p 133-135 °C. Yield: 60%. **^{1}H NMR** (500 MHz, $CDCl_3$) δ 7.54 (d, *J* = 8.1 Hz, 1H), 7.47 (d, *J* = 7.8 Hz, 1H), 7.31 – 7.27 (m, 1H), 7.20 (t, *J* = 7.4 Hz, 1H), 5.69 (s, 1H), 5.66 (d, *J* = 7.3 Hz, 1H), 3.62 (dddd, *J* = 15.1, 13.2, 5.8, 2.5 Hz, 1H), 3.08 – 2.98 (m, 1H), 2.87 (dd, *J* = 16.6, 2.7 Hz, 1H), 2.70 – 2.64 (m, 1H), 2.62 – 2.58 (m, 1H), 2.49 (dq, *J* = 14.2, 2.4 Hz, 1H), 2.43 (dd, *J* = 12.9, 3.1 Hz, 1H), 2.31 (tt, *J* = 12.9, 4.1 Hz, 1H), 2.24 (ddd, *J* = 14.0, 4.5, 2.1 Hz, 1H) , 2.01 (dd, *J* = 14.0, 3.6 Hz, 1H), 1.97 – 1.83 (m, 2H). **^{13}C{^{1}H} NMR** (125 MHz, $CDCl_3$) δ 175.1, 135.4, 128.5, 127.8, 123.2, 121.2, 120.9, 118.6, 111.0, 110.7, 79.2, 43.0, 40.4, 39.4, 35.3, 31.4, 28.0, 25.3, 23.3. **HRMS** (DART) m/z calcd for $C_{19}H_{20}N_3O_2$ $[M + H]^+$: 322.1555, found 322.1542. **FT-IR** (ATR): ν (cm^{-1}) 3352, 3058, 2927, 2242, 1730, 1644, 1565, 1462, 1272, 1068, 744. Structural assignments were confirmed with additional 2D NMR data (gHSQC).

2.4 Procedures for the synthesis of ketone 12.

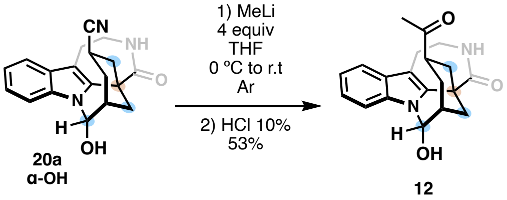

Scheme S3. Synthesis of Ketone **12**.

In a round-bottom flask equipped with a magnetic stir bar, **20a** (0.093 mmol, 1.0 equiv) was dissolved in dry THF (1.9 mL, 0.05 M) and cooled to 0 °C under an argon atmosphere. Methyllithium (1.6 M in diethyl ether, 0.23 mL, 0.373 mmol) was then added dropwise via syringe. Caution: this reagent is highly moisture- and air-sensitive and reacts violently with water; appropriate safety precautions should be taken. After 2 h, the reaction mixture was quenched with a saturated aqueous solution of ammonium chloride and extracted with DCM (3 × 30 mL). The combined organic phase was dried over Na_2SO_4, filtered and the solvent was removed under reduced pressure. The reaction crude was purified by column chromatography 96:4 DCM:MeOH to give **12** (0.017 g, 0.05 mmol) as a white solid, m.p 154-156 °C. Yield: 53 %. **^{1}H NMR** (500 MHz, Acetone-*d6*) δ 7.52 (dt, *J* = 8.1, 0.9 Hz, 1H), 7.45 (dt, *J* = 7.8, 1.0 Hz, 1H), 7.15 (ddd, *J* = 8.2, 7.1, 1.2 Hz, 1H), 7.07 (ddd, *J* = 7.9, 7.0, 1.0 Hz, 1H), 5.90 (d, *J* = 7.1 Hz, 1H), 5.19 (d, *J* = 7.1 Hz, 1H), 3.89 (tdd, *J* = 13.0, 5.6, 2.8 Hz, 1H), 3.53 (dddd, *J* = 15.0, 7.9, 4.0, 3.2 Hz, 1H), 3.04 (dt, *J* = 16.3, 2.6 Hz, 1H), 2.87 (ddd, *J* = 16.5, 12.9, 4.0 Hz, 1H), 2.58 (ddd, *J* = 6.3, 4.0, 2.3 Hz, 1H), 2.53 (ddd, *J* = 12.7, 4.0, 2.0 Hz, 1H), 2.48 (dtd, *J* = 13.7, 2.9, 1.8 Hz, 1H), 2.32 (dt, *J* = 12.9, 4.1 Hz, 1H), 2.31 – 2.21 (m, 1H), 2.07 (d, *J* = 3.6 Hz, 1H), 2.01 (s, 3H), 1.74 (dd, *J* = 13.3, 5.1 Hz, 1H), 1.68 (t, *J* = 12.5 Hz, 1H). **^{13}C{^{1}H} NMR** (125 MHz, Acetone- *d6*) δ 209.8, 176.1, 136.8, 131.5, 129.6, 122.5, 120.6, 118.8, 111.9, 111.1, 79.9, 45.5, 44.5, 41.1, 39.6, 37.5, 37.5, 31.1, 28.1, 27.0. **HRMS** (DART) m/z calcd for $C_{20}H_{23}N_2O_3$ $[M + H]^+$: 339.1708, found 339.1712. **FT-IR** (ATR): ν (cm^{-1}) 3189, 3056, 2928,1716, 1655, 1462, 739.

2.5 Procedures for the synthesis of 21.

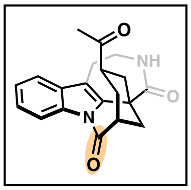

Pyridinium dichromate (PDC, 0.028 g, 0.073 mmol) was added in two portions over 30 min to a solution of **12** (0.017 g, 0.050 mmol) in dry DCM (1.0 mL, 0.05 M) at room temperature under a N_2 atmosphere. The reaction mixture was stirred for 2 h, then filtered through Celite and washed with EtOAc. The solvent was removed under reduced pressure, and the residue was purified by column chromatography 96:4 DCM:MeOH to give **21** (0.008 g, 0.024 mmol) as a white solid, m.p 247 °C (dec). Yield: 47 %. **^{1}H NMR** (400 MHz, $CDCl_3$) δ 8.54 (dt, *J* = 8.3, 0.9 Hz, 1H), 7.45 (ddd, *J* = 7.7, 1.5, 0.8 Hz, 1H), 7.42 – 7.38 (m, 1H), 7.34 (dd, *J* = 7.5, 1.3 Hz, 1H), 6.31 (t, *J* = 6.1 Hz, 1H), 3.93 – 3.85 (m, 1H), 3.55 – 3.48 (m, 1H), 3.28 – 3.19 (m, 1H), 3.04 – 2.94 (m, 2H), 2.50 – 2.37 (m, 4H), 2.33 (dd, *J* = 13.8, 2.8 Hz, 1H), 2.10 (s, 3H), 1.98 (t, *J* = 13.4 Hz, 1H), 1.84 – 1.74 (m, 1H). **^{13}C{^{1}H} NMR** (100 MHz, $CDCl_3$) δ 208.5, 174.7, 171.5, 134.4, 130.9, 129.8, 125.8, 124.3, 118.1, 117.9, 116.6, 44.8, 43.6, 40.1,

39.4, 37.3, 34.0, 30.2, 28.1, 26.0. **HRMS** (DART) m/z calcd for $C_{20}H_{21}N_2O_3$ $[M + H]^+$: 337.1470, found 337.1509. **FT-IR** (ATR): ν (cm^{-1}) 3197, 2919, 1708, 1686, 1661, 1455, 1374, 751.

Table S2. Reaction Conditions for the Oxidation of Dihydrotronocarpine.

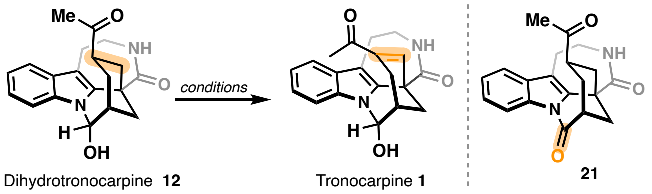

Entry	Conditions	T (h)	Isolated Compound (%)
1	1) LHMDS (1 equiv), TMSCl (1 equiv), 0 °C 2) Pd(OAc)$_2$ (1 equiv)	6	**21** (isolated in traces)
2	1) TEA (3.4 equiv) , NaI (3.75 equiv), TMSCl (3.4 equiv), MeCN, rt 2) Pd(OAc)$_2$ (3.5 equiv), MeCN, rt	24	**21** (isolated in traces)
3	IBX/DMSO	12	----------------
4	Jørgensen Catalyst DDQ, THF	24	----------------
5	Benzylamine, Pd(OAc)$_2$, O_2 DMSO, 75 °C	24	----------------
6	Phosphoric acid, DDQ, Benzene	24	**21** (isolated in traces)
7	PDC (1.5 equiv), DCM, rt	2	**21** (47)

3. Experimental procedures

^{1}H NMR (500 MHz, $CDCl_3$) and **$^{13}C\{^{1}H\}$ NMR** (125 MHz, $CDCl_3$) **for compound 14.**

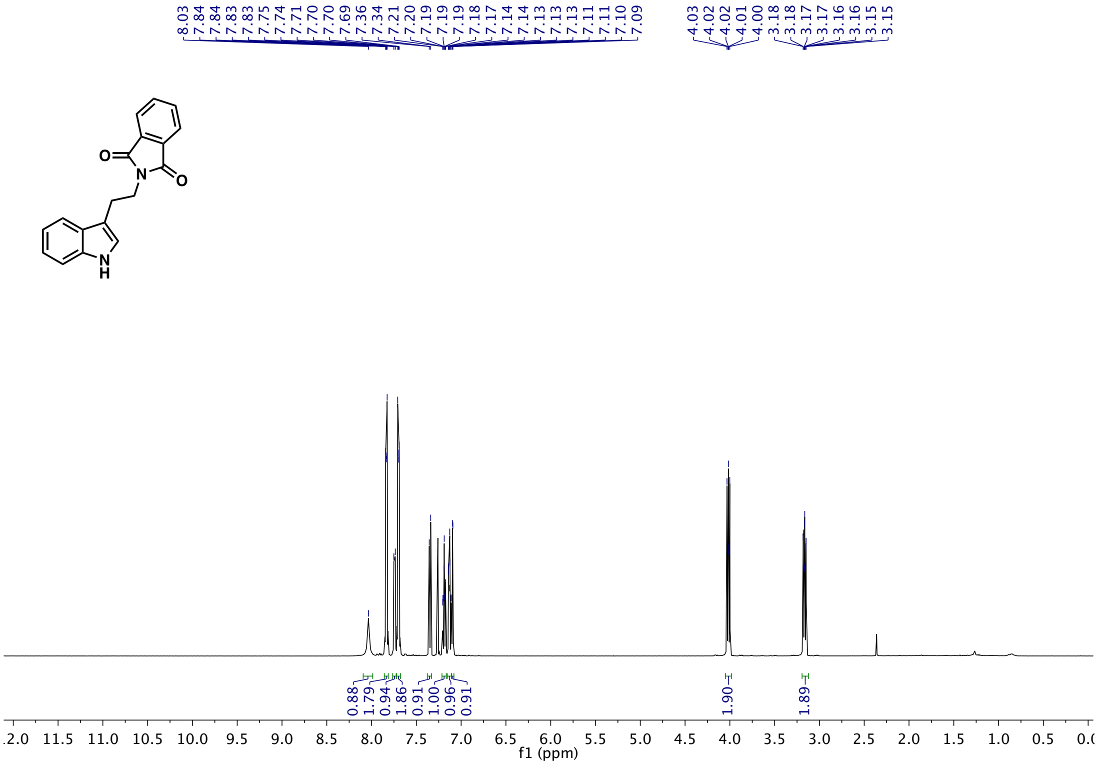

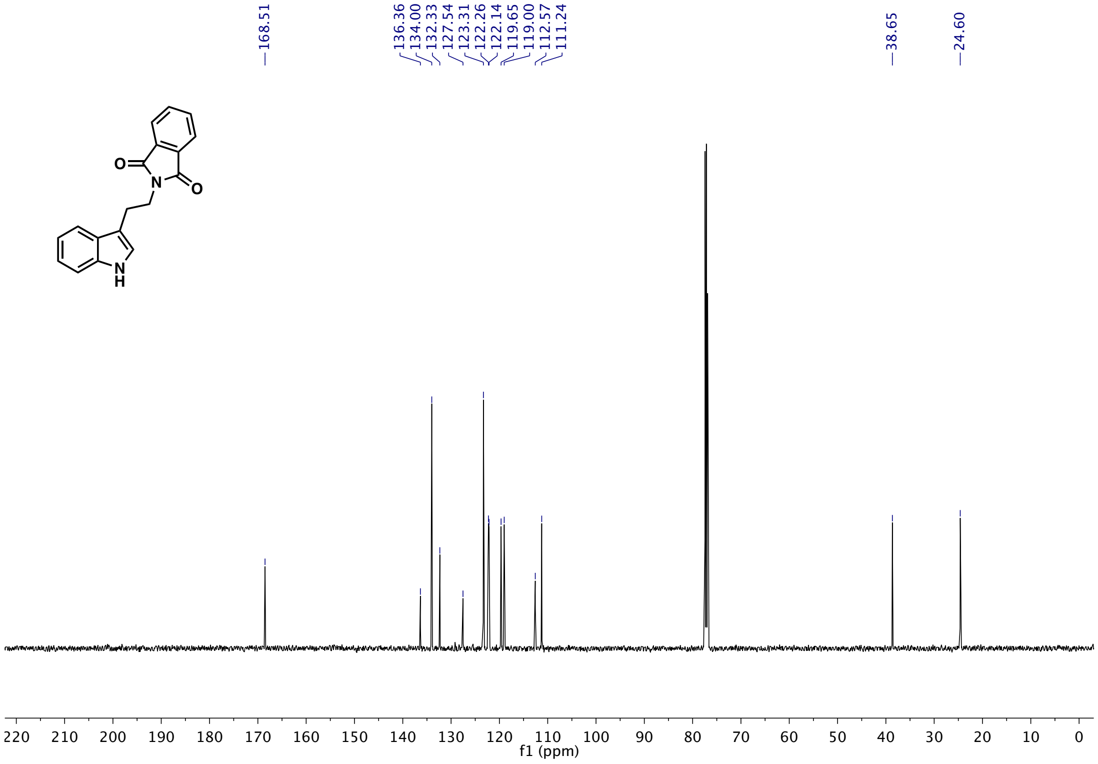

^{1}**H NMR** (500 MHz, $CDCl_3$) and ^{13}**C{^{1}H} NMR** (125 MHz, $CDCl_3$) **for compound S3.**

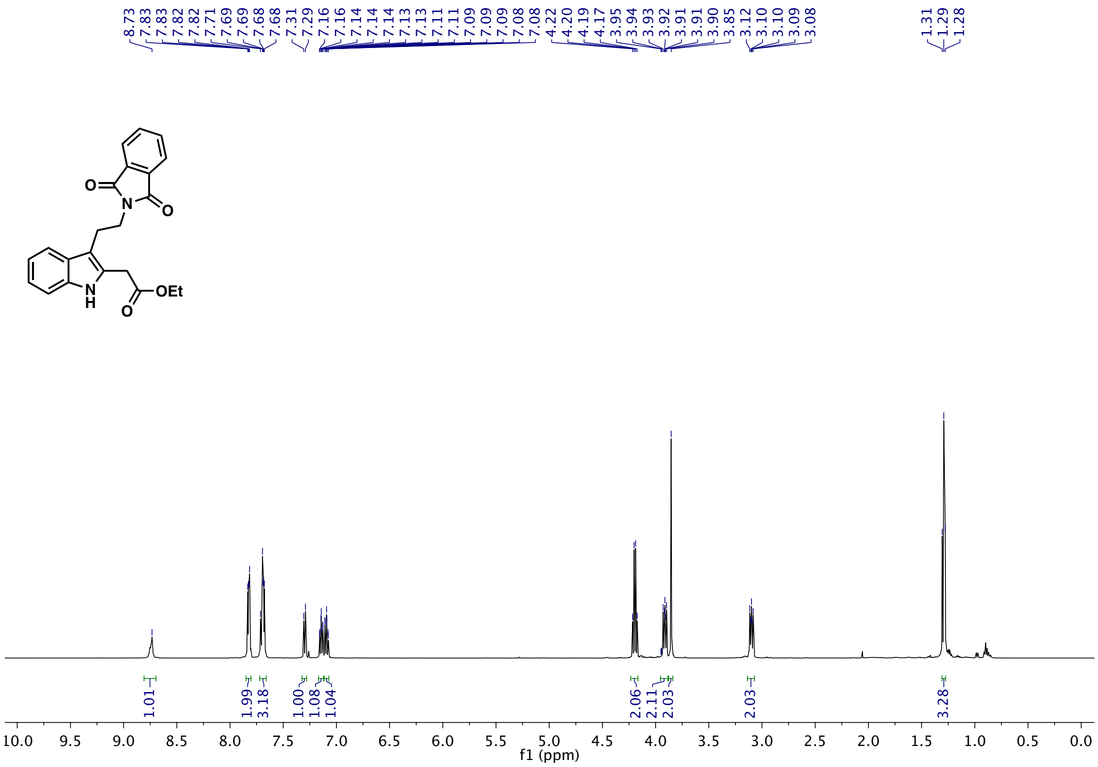

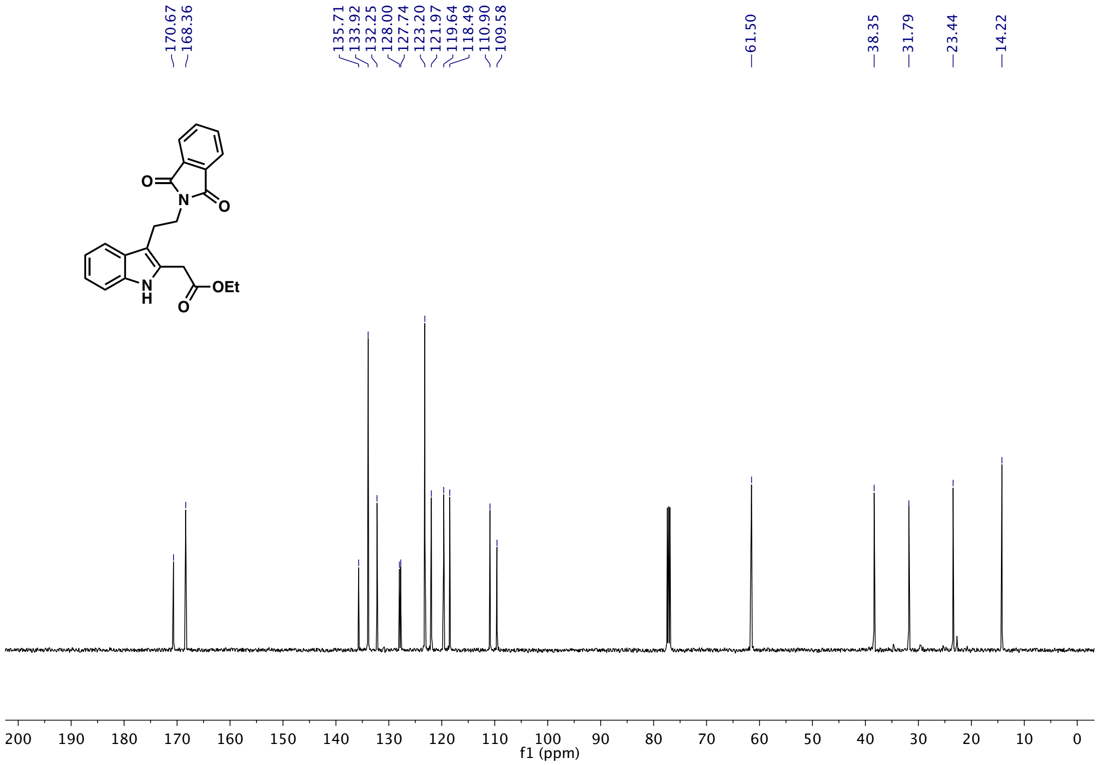

^{1}H NMR (500 MHz, DMSO-*d6*) and **^{13}C{^{1}H} NMR** (125 MHz, DMSO-*d6*) **for compound 9.**

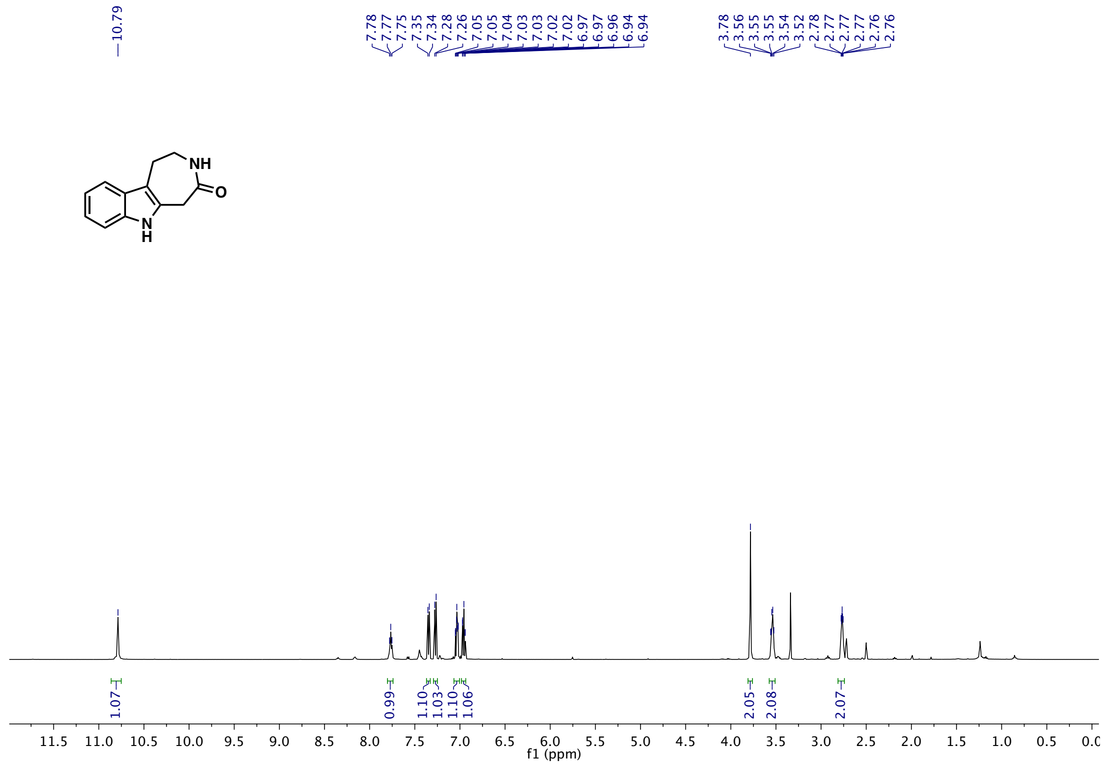

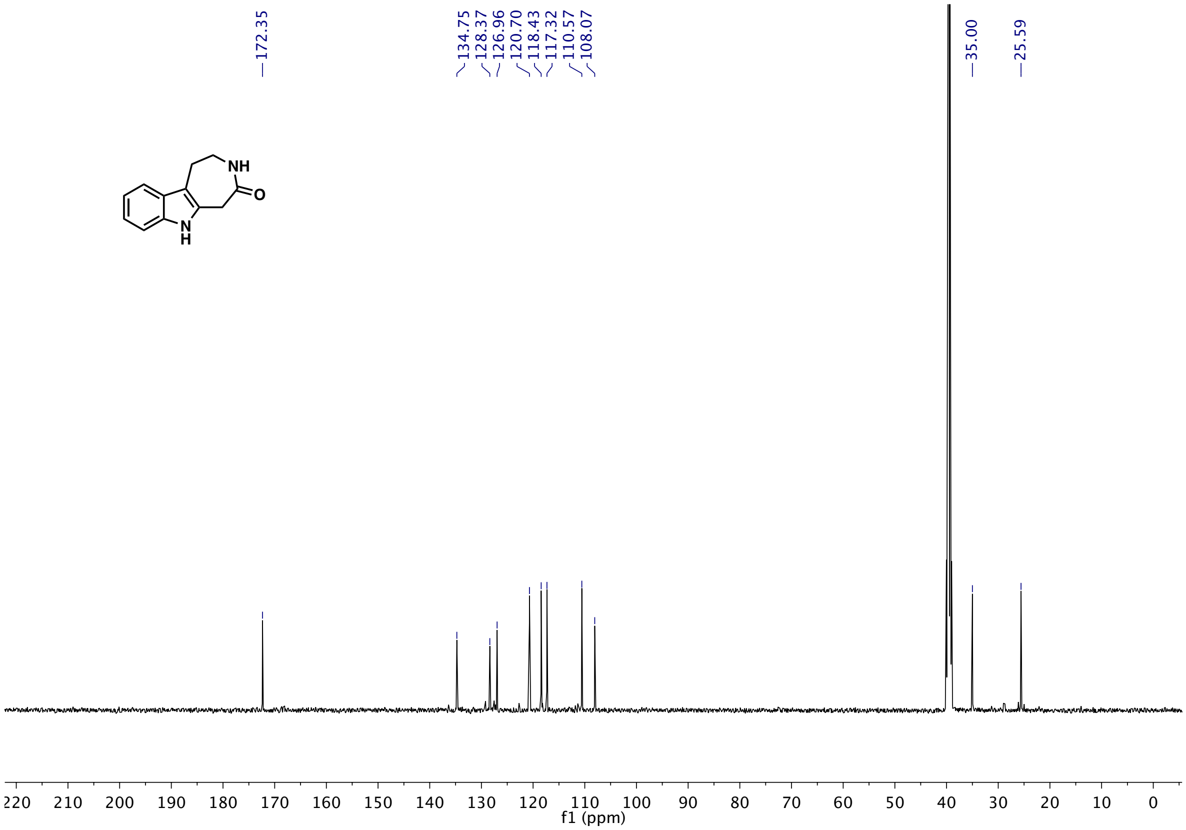

^{1}H NMR (700 MHz, $CDCl_3$) and **$^{13}C\{^{1}H\}$ NMR** (175 MHz, $CDCl_3$) **for compound 13.**

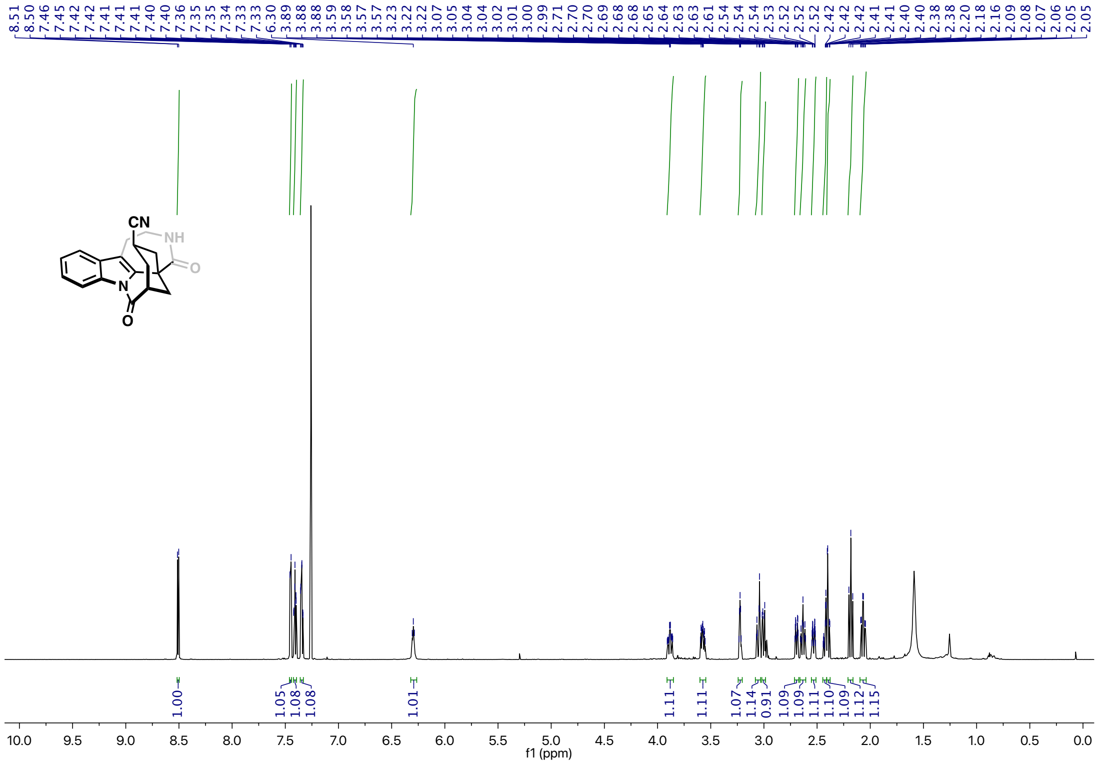

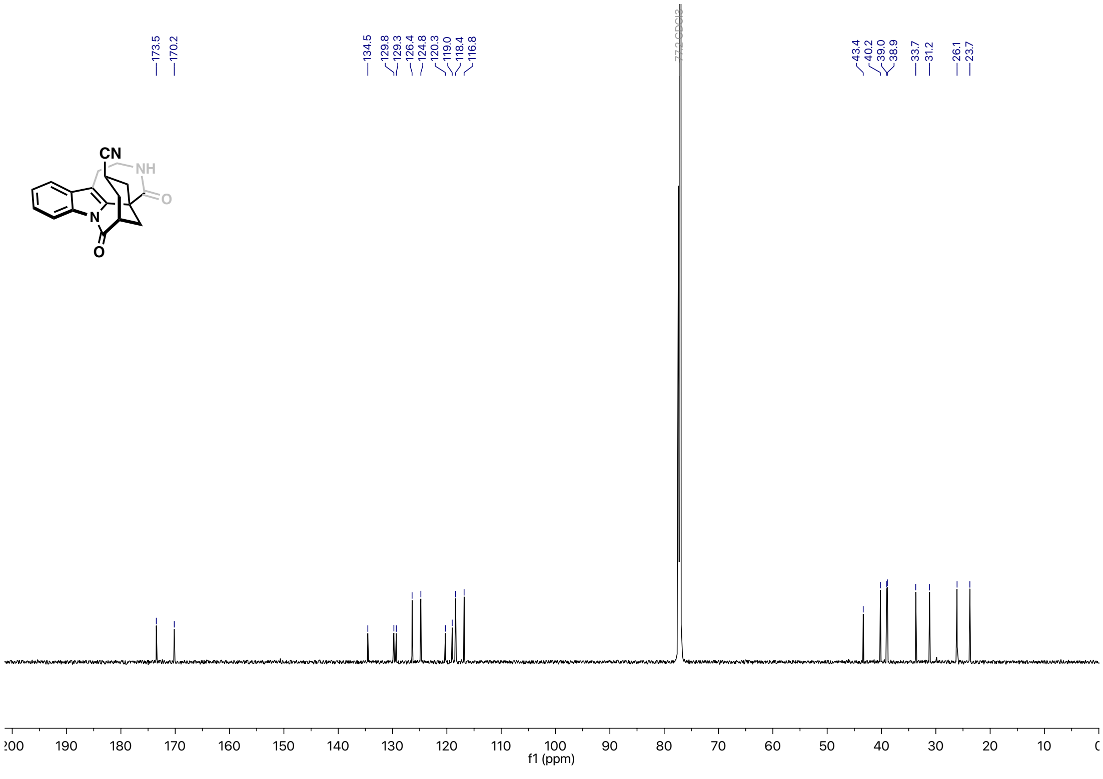

^{1}H NMR (500 MHz, DMSO-*d6*) and **$^{13}C\{^{1}H\}$ NMR** (125 MHz, DMSO-*d6*) **for compound 20b.**

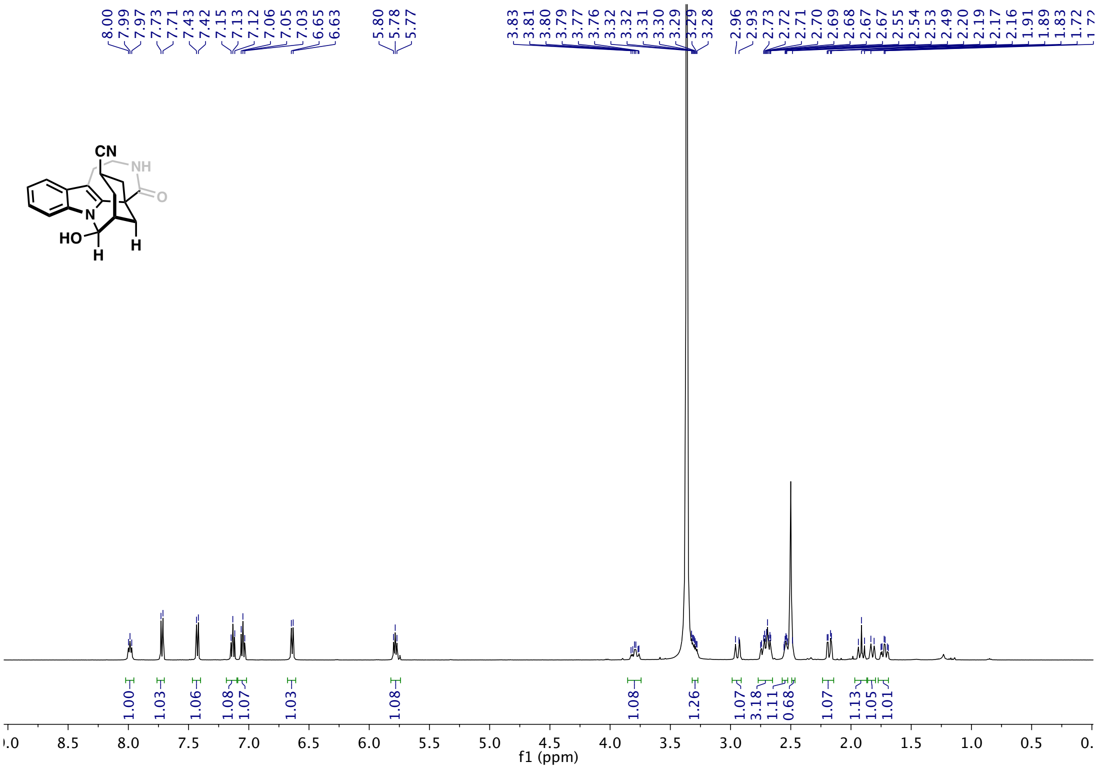

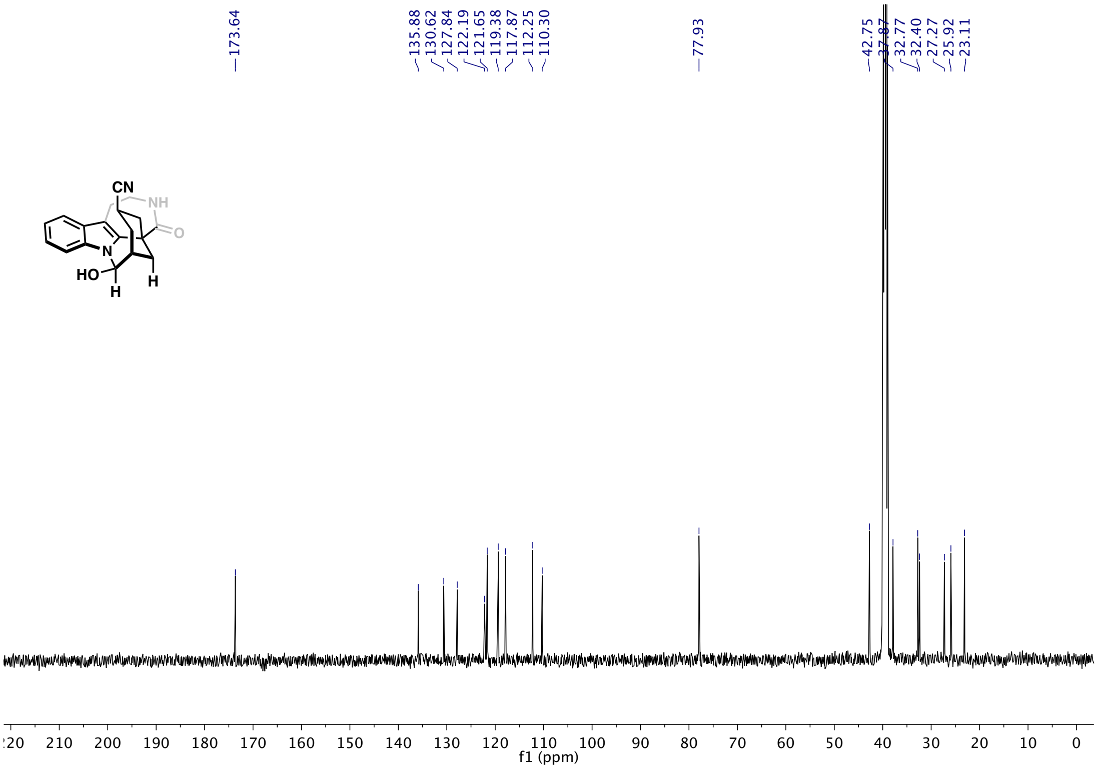

HSQC for compound 20b.

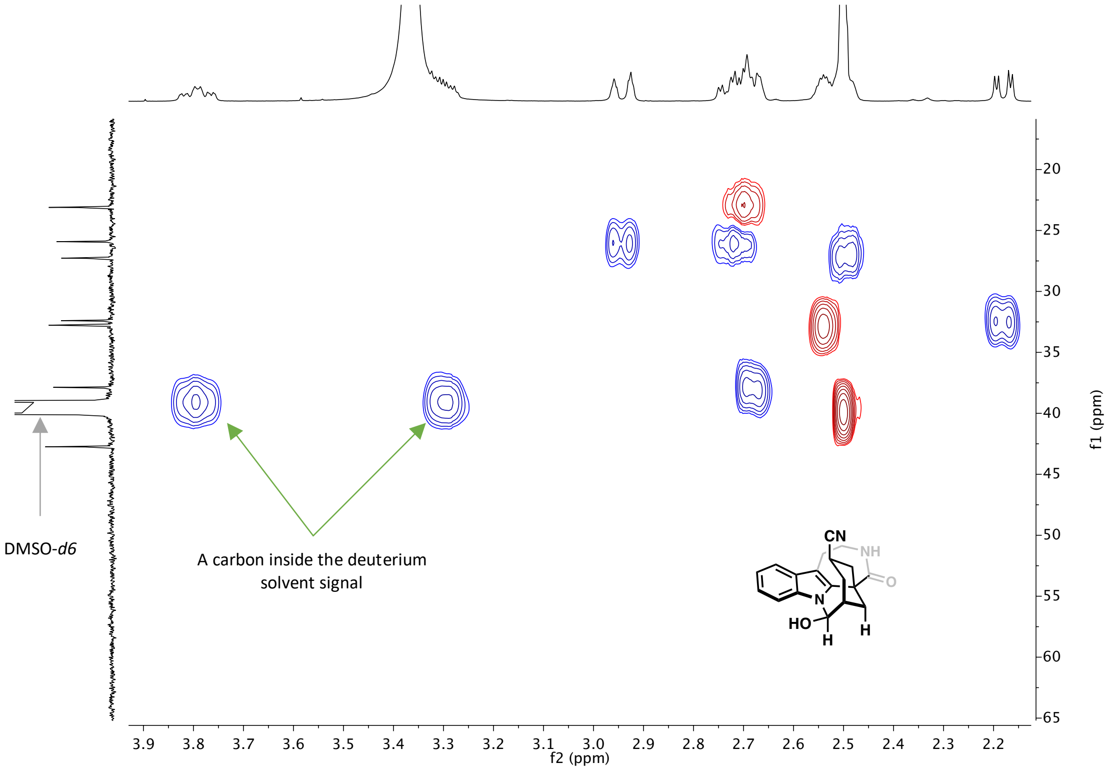

ROESY for compound 20b.

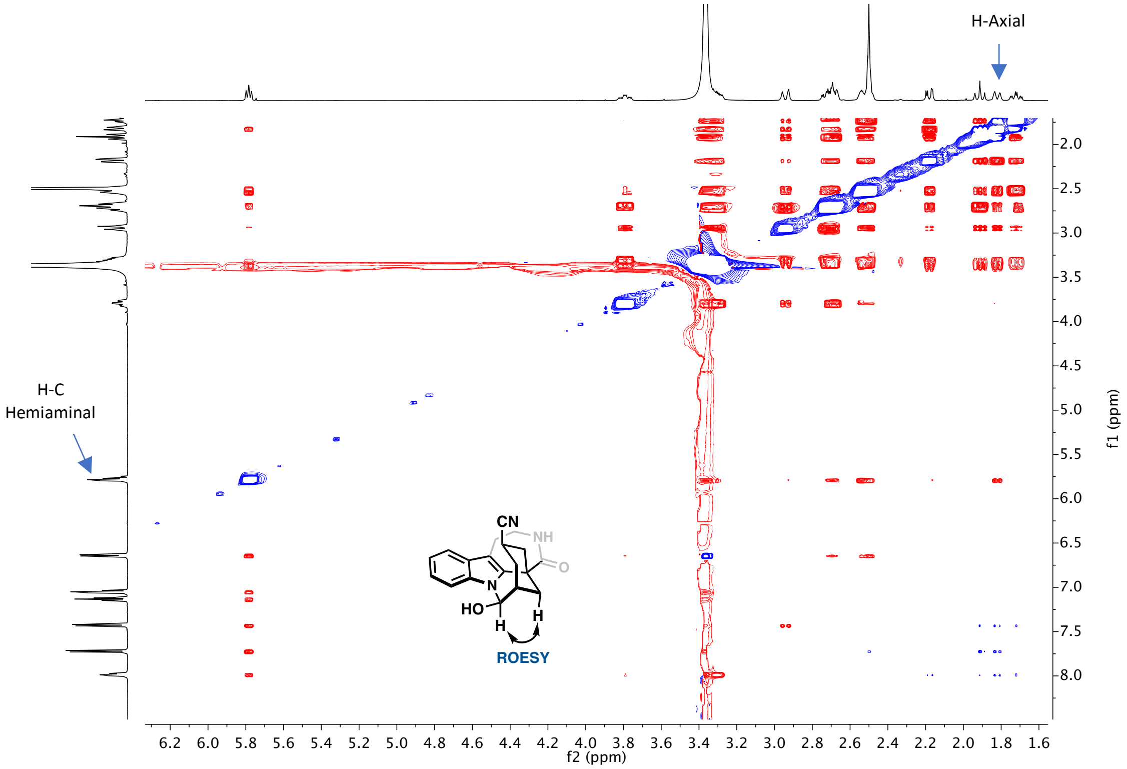

^{1}H NMR (500 MHz, $CDCl_3$) and **^{13}C{^{1}H} NMR** (125 MHz, $CDCl_3$) **for compound 20a.**

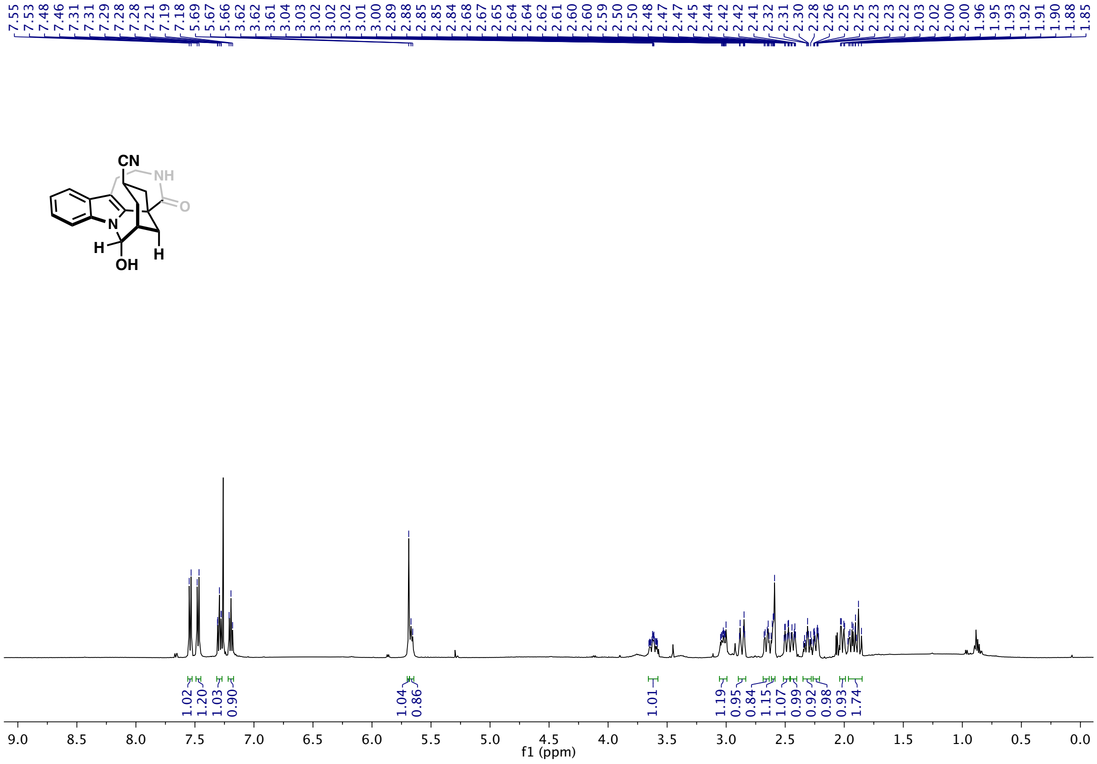

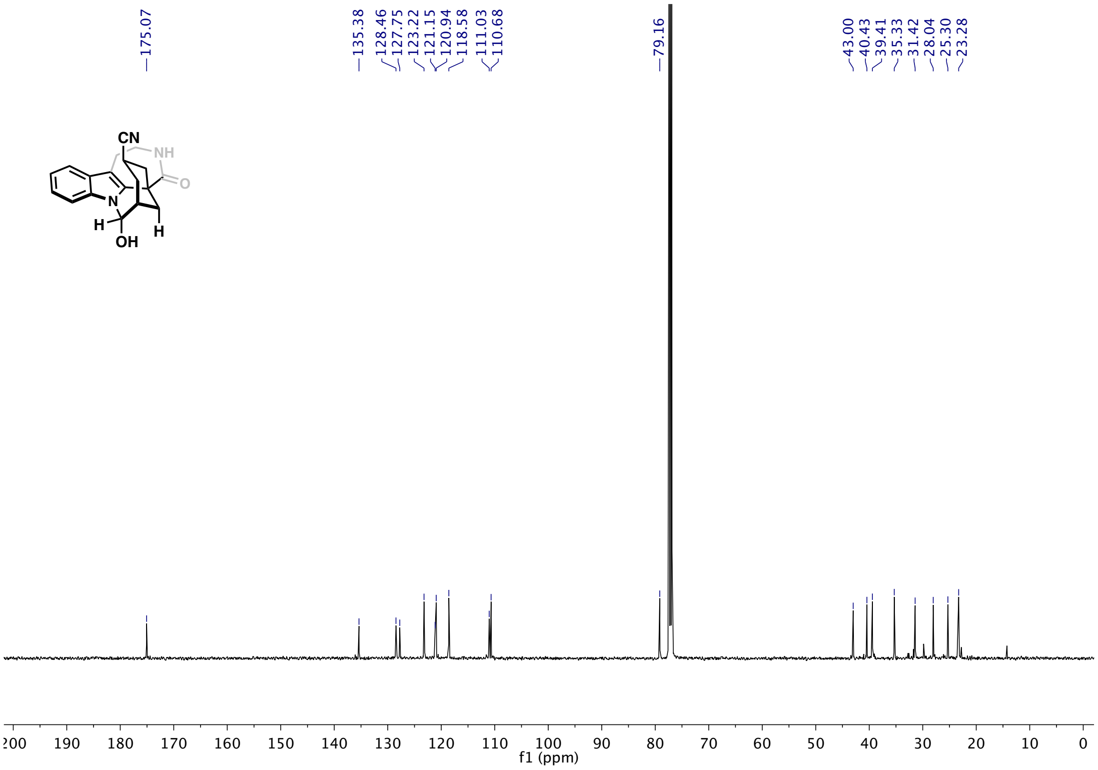

HSCQ for compound 20a.

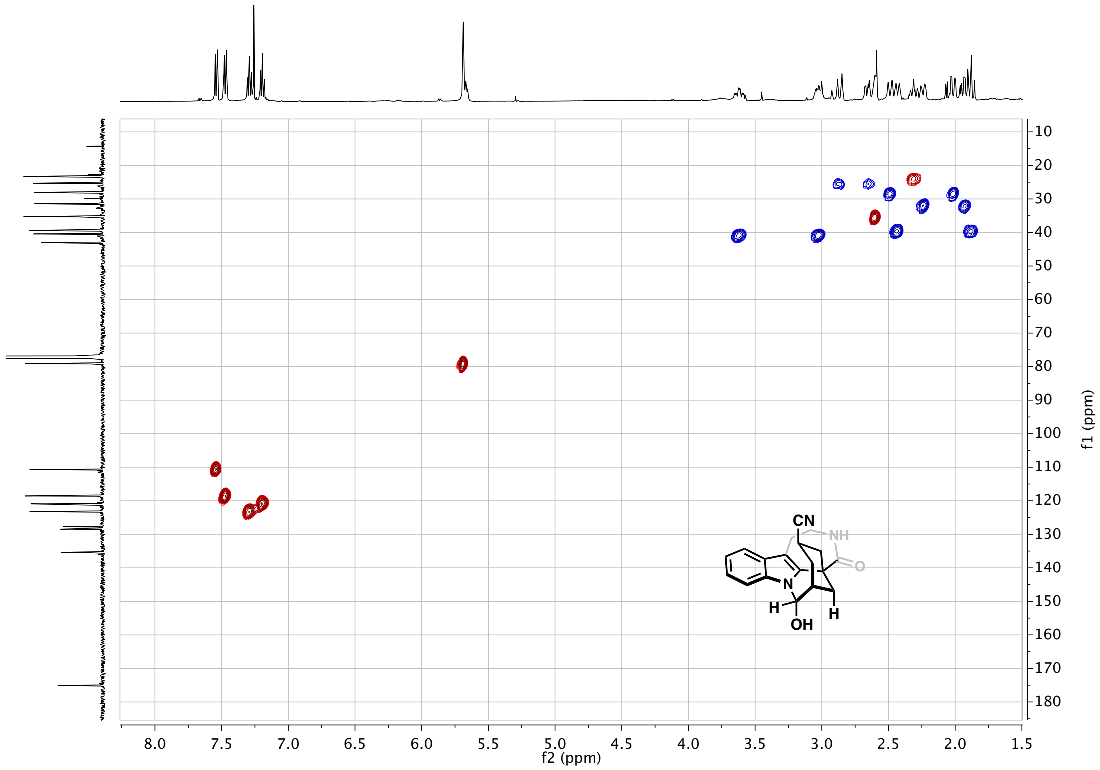

¹H NMR (500 MHz, Acetone- *d6*) and **¹³C{¹H} NMR** (125 MHz, Acetone- *d6*) **for compound 12.**

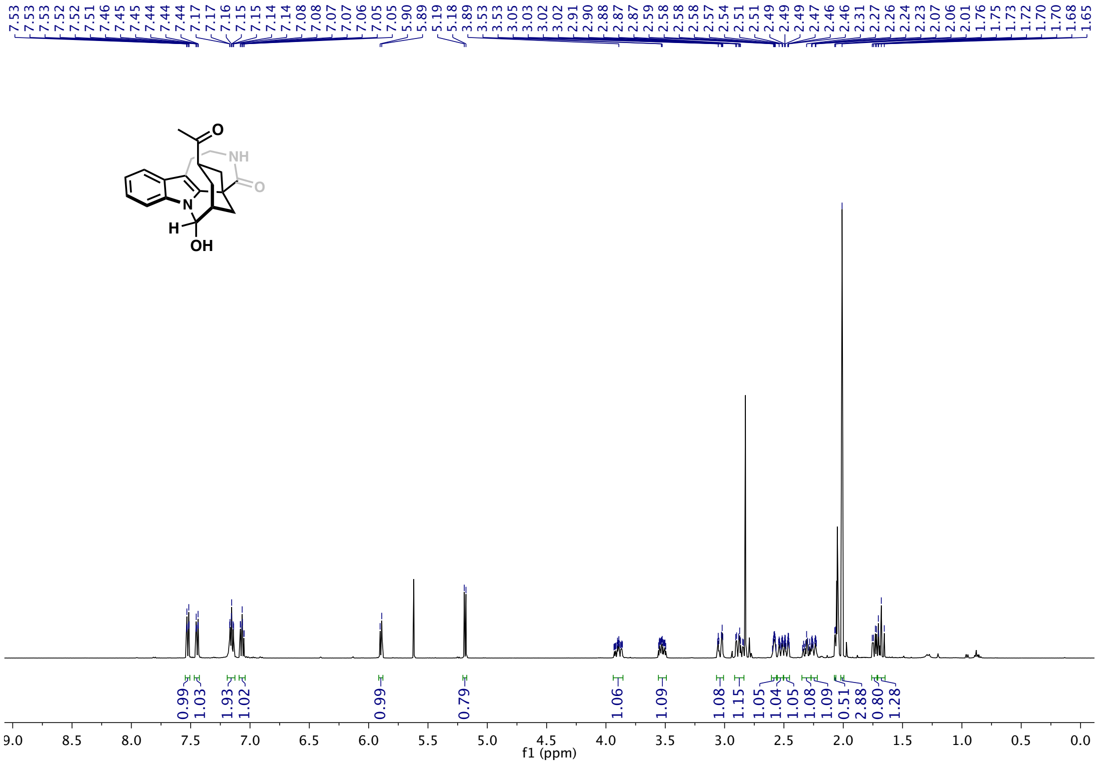

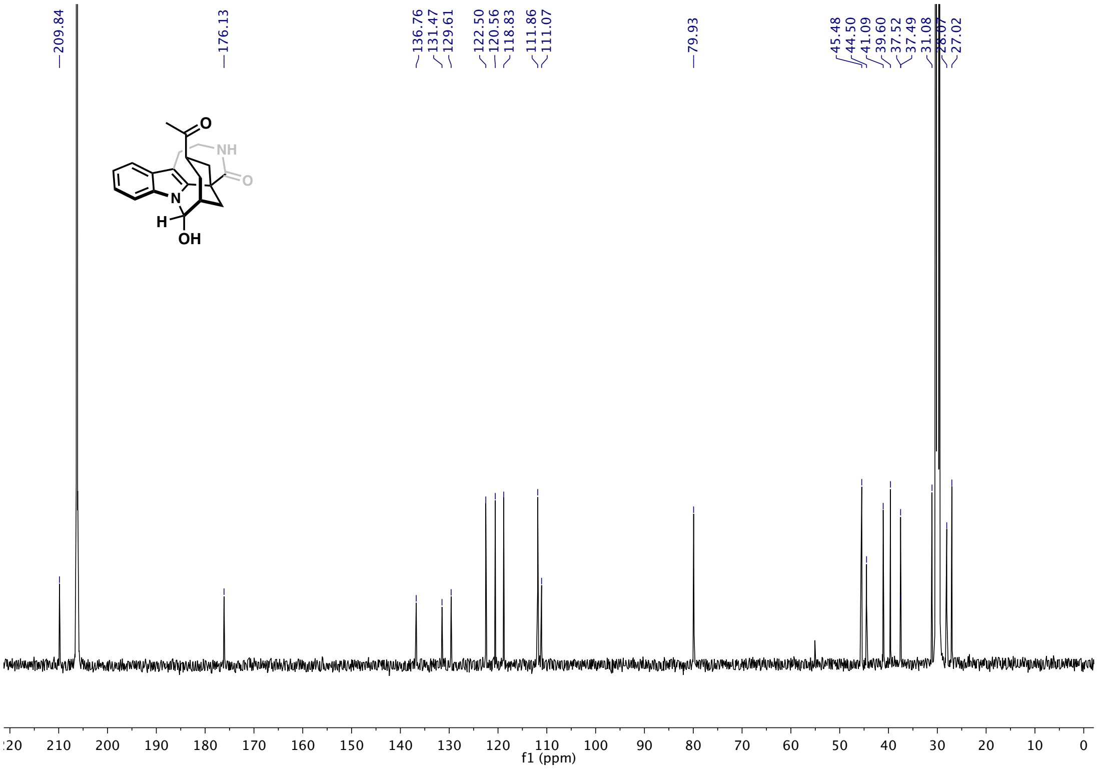

^{1}H NMR (400 MHz, $CDCl_3$) and **^{13}C{^{1}H} NMR** (100 MHz, $CDCl_3$) **for compound 21.**

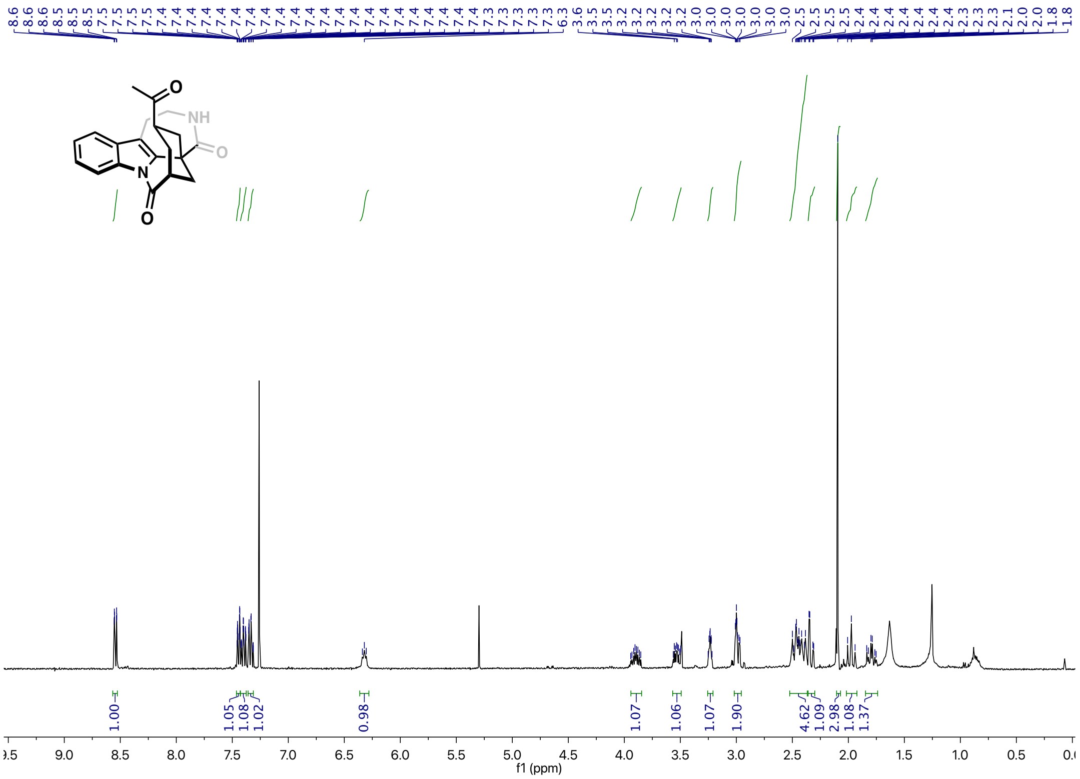

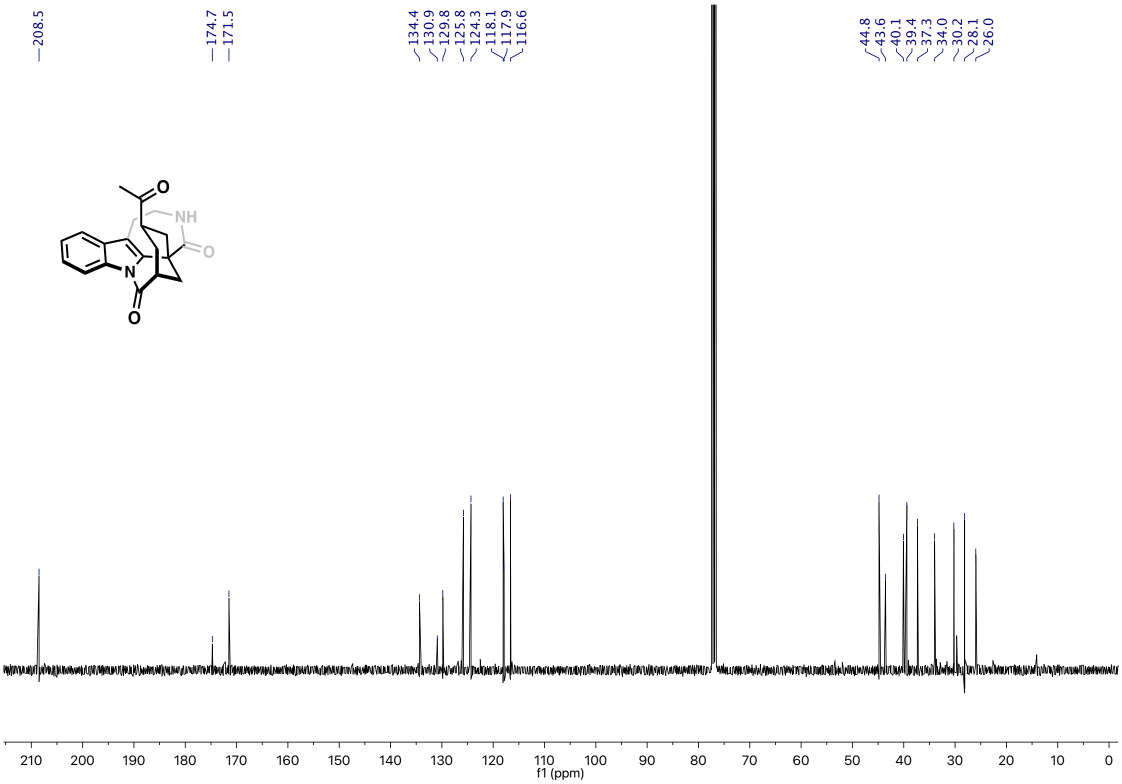

4. X-Ray Crystallographic Data

Table 2. Crystal data and structure refinement for **compound 20a**. Ellipsoid contour % probability: 50%. (CCDC 2480440).
The compound crystallizes with undetermined solvent and was refined with platon squeeze program.

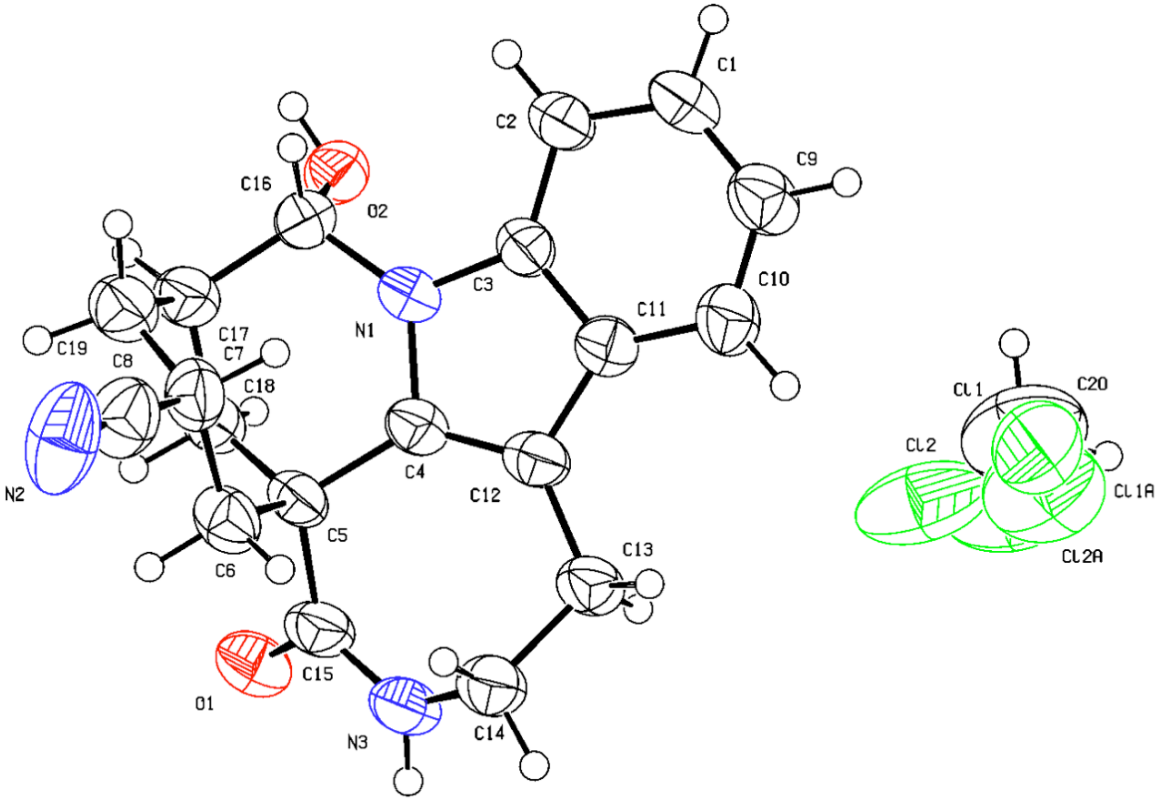

Identification code	mo_051MGL24_sq	
Empirical formula	C39 H40 Cl2 N6 O4	
Formula weight	727.67	
Temperature	150(2) K	
Wavelength	0.71073 Å	
Crystal system	Trigonal	
Space group	R-3	
Unit cell dimensions	a = 18.5456(3) Å	a= 90°.
	b = 18.5456(3) Å	b= 90°.
	c = 30.9336(7) Å	g = 120°.
Volume	9213.9(4) Å3	
Z	9	
Density (calculated)	1.180 Mg/m3	

Absorption coefficient	0.203 mm-1
F(000)	3438
Crystal size	0.250 x 0.193 x 0.150 mm3
Theta range for data collection	2.196 to 27.493°.
Index ranges	-23<=h<=22, -24<=k<=21, -31<=l<=40
Reflections collected	22230
Independent reflections	4681 [R(int) = 0.0574]
Completeness to theta = 25.242°	99.6 %
Absorption correction	None
Refinement method	Full-matrix least-squares on F2
Data / restraints / parameters	4681 / 49 / 267
Goodness-of-fit on F2	2.821
Final R indices [I>2sigma(I)]	R1 = 0.1811, wR2 = 0.4956
R indices (all data)	R1 = 0.2187, wR2 = 0.5553
Extinction coefficient	0.0040(15)
Largest diff. peak and hole	1.974 and -0.854 e.Å-3

5. References

1. Feng, P.; Fan, Y.; Xue, F.; Liu, W.; Li, S.; Shi, Y. An Approach to the Hexacyclic Skeleton of Trigonoliimines. *Org. Lett*. **2011**, *13*, 5827–5829.
2. Reyes-Gutiérrez, P. E.; Torres-Ochoa, R. O.; Martínez, R.; Miranda, L. D. Synthesis of Azepino[4,5-*b*]indolones *Via* an Intermolecular Radical Oxidative Substitution of *N*-Boc Tryptamine**.** *Org. Biomol. Chem.* **2009**, *7*, 1388–1396.
